# The Neural System of Postdecision Evaluation in Rostral Frontal Cortex during Problem-solving Tasks

**DOI:** 10.1523/ENEURO.0188-16.2016

**Published:** 2016-08-29

**Authors:** Xiaohong Wan, Kang Cheng, Keiji Tanaka

**Affiliations:** 1 Cognitive Brain Mapping Laboratory, RIKEN Brain Science Institute, Wako, Saitama 351-0198, Japan; 2 State Key Laboratory of Cognitive Neuroscience and Learning and IDG/McGovern Institute for Brain Research, Beijing Normal University, Beijing, 100875, China, and; 3 Support Unit for Functional Magnetic Resonance Imaging, RIKEN Brain Science Institute, Wako, Saitama 351-0198, Japan

**Keywords:** dorsal anterior cingulate cortex, expert, fMRI, frontopolar cortex, metacognition, shogi

## Abstract

Little attention has been paid to the postdecision processing in fMRI studies with task paradigms in which there was no explicit feedback. Although late-onset BOLD responses were previously observed in the lateral frontopolar cortex after the familiar-novel decision on visually presented words, the nature of neural activations that caused the late-onset BOLD responses remained elusive. We here found, in human experts conducting complicated problem-solving tasks in their expertise domain, that the rostral frontal cortex, including the lateral frontopolar cortex, along with the anterior inferior parietal lobule, was activated only during the postdecision period, although there was no feedback. That is, these areas showed late-onset BOLD responses, and fitting of the BOLD responses with different models indicates that they were caused by neural activations that occurred after the decision. However, there was no response after performing a sensory-motor control task, and the magnitude of postdecision activations was correlated with the degree of uncertainty about the preceding decision, which suggests that the postdecision neural activations were associated with the preceding decision procedure. Furthermore, the same set of areas was more strongly activated when the subject explicitly rethought the preceding problem. These results suggest that the rostral frontal cortex, together with anterior inferior parietal lobule, comprises a network for uncertainty monitoring and exploration of alternative resolutions in postdecision evaluation. The present results thus introduce a new aspect of the functional gradient along the rostrocaudal axis in the frontal cortex.

## Visual Abstract

**Figure:**
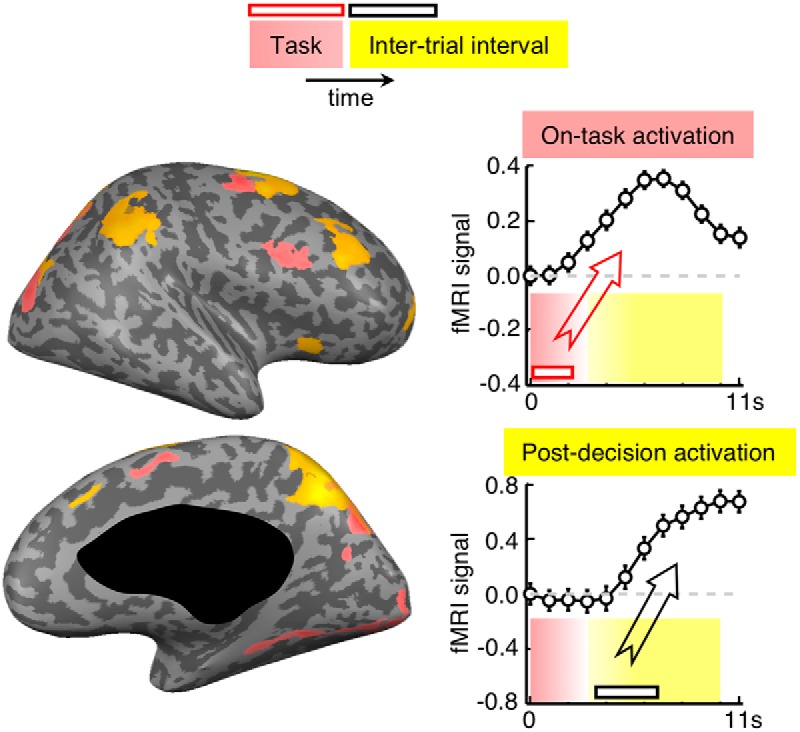

## Significance Statement

After generating and selecting a solution for a given problem, we often evaluate the solution, even without explicit feedback. This may be to change the solution when there is an opportunity for change, and, more generally, to deepen our understanding of similar problems. By using checkmate problems in Japanese chess, shogi, without giving any explicit feedback, we here found that the postdecision evaluation is mainly conducted by a frontoparietal network involving rostral frontal areas. These findings also introduce a new aspect of functional gradient in frontal cortex: postdecision evaluation in rostral areas and online task execution in caudal areas.

## Introduction

While there is a consensus that the frontal cortical areas anterior to the primary motor cortex play essential roles in cognitive control, our knowledge about how these areas are functionally organized remains limited. Consistent with the rostrocaudal gradient in their intrinsic and external anatomical connections (Barbas and Pandia, 1991; Carmichael and Price, 1996; Fuster, 1997; Petrides, 2005; Saleem et al., 2014), neuroimaging and neuron-recording studies have found evidence suggesting a functional gradient in this region. Rostral frontal areas are more involved in domain-general processing with longer time span and execution of tasks with higher-order structure, whereas caudal frontal areas are more involved in domain-specific processing with shorter time span and execution of simpler tasks (Fuster, 1997; Ramnani and Owen, 2004; Botvinick, 2007; Koechlin and Summerfield, 2007; Badre and D’Esposito, 2009). The interpretation of these previous results, however, requires caution, as most of previous studies used deterministic tasks with limited problem space. The problems in real life are more complex and are usually accompanied with uncertainty; thus, exploration is required to resolve the uncertainty for taking the currently optimal action (Yoshida and Ishii, 2006; Badre et al., 2012). The rostrocaudal gradient may be associated with the control distribution between exploration and exploitation. Indeed, recent neuroimaging studies have suggested specific involvement of rostral frontal areas in exploration of nondefault options (Daw et al., 2006; Boorman et al., 2009, 2013; Kolling et al., 2012, 2014).

In the present study, we explored the possibility that the rostrocaudal gradient in frontal cortex is differentially associated with online control of task execution and postdecision process of high-level monitoring as well as exploration of alternatives. Although the classical cognitive theory of human problem-solving proposes that decision-making is generally followed by evaluation (Newell and Simon, 1972; Engel et al., 1993; Zelazo et al., 1997), neural correlates of the postdecision evaluation have been little examined, except for the cases in which decisions were followed by explicit feedback. Although late-onset BOLD signals are observed in the lateral frontopolar cortex (lFPC) in the tasks requiring familiar-versus-novel judgment on visually presented words (Schacter et al., 1997; Buckner et al, 1998; Reynolds et al., 2006), the association of the late-onset BOLD signals with postdecision neural activities remains elusive.

We measured brain activities of expert players of shogi, Japanese chess, while they generated an idea of the best next-move in a given board position. No feedback was given after decisions were made. We found that caudal frontal areas were activated only during the online processing of the generation task. In contrast, specific activations were observed after decisions in rostral frontal areas, including the lFPC, dorsal anterior cingulate cortex (dACC), and middle dorsolateral prefrontal cortex (mDLPFC), and in the anterior inferior parietal lobule (aIPL). We also found that postdecision activities tended to be larger when the subject was more uncertain about the decision that the subject had just made, and the same group of areas was activated when the subjects rethought the problem. It is reasonable to assume that uncertainty about the preceding decision drove people to explore other possible solutions in rethinking. The present results suggest that postdecision evaluation, which may be driven by decision uncertainty and be associated with exploration, takes place primarily in the frontoparietal network, including rostral frontal areas.

## Materials and Methods

### Subjects

All the subjects were right-handed Japanese males. Experiments 1 and 2 were conducted on 17 professional players (30.2 ± 1.5 years old) and 17 high-rank amateur players (proficient level: 2-4 dan, 32.5 ± 2.3 years old). Another group of 17 high-rank amateur players (3-4 dan, 31.4 ± 2.7 years old) participated in Experiment 3. Nineteen novice subjects (20.3 ± 0.2 years old) participated in Experiment 4. Informed consent was obtained from each subject in accordance with protocols approved by the Institute Research Ethics Committee of RIKEN.

### Tasks

Experiments 1 and 2 were originally designed to reveal neural substrates of quick next-move generation in expert players. Brain activities specifically associated with quick next-move generation, in contrast to those associated with deliberative search, had been reported (Wan et al., 2011). We made unexpected findings in these experiments, that is, the post-task activations in the post-task network common to the quick generation and deliberative search, which is the main subject of the present paper. To further examine properties of the post-task activations, we designed Experiment 3. Experiment 4 was originally designed to study the development of the capability of quick next-move generation and of associated brain activities along a long-term training of the game skill. The main results of Experiment 4 have been previously reported (Wan et al., 2012). The development of the post-task activations along the training is reported in the present paper.

Subjects viewed the images for the tasks through an optic-fiber goggle system (resolution 800 × 600). All visual stimuli (200 × 200 pixels) were restricted to within 3 degrees of visual angle.

**Figure 1.**
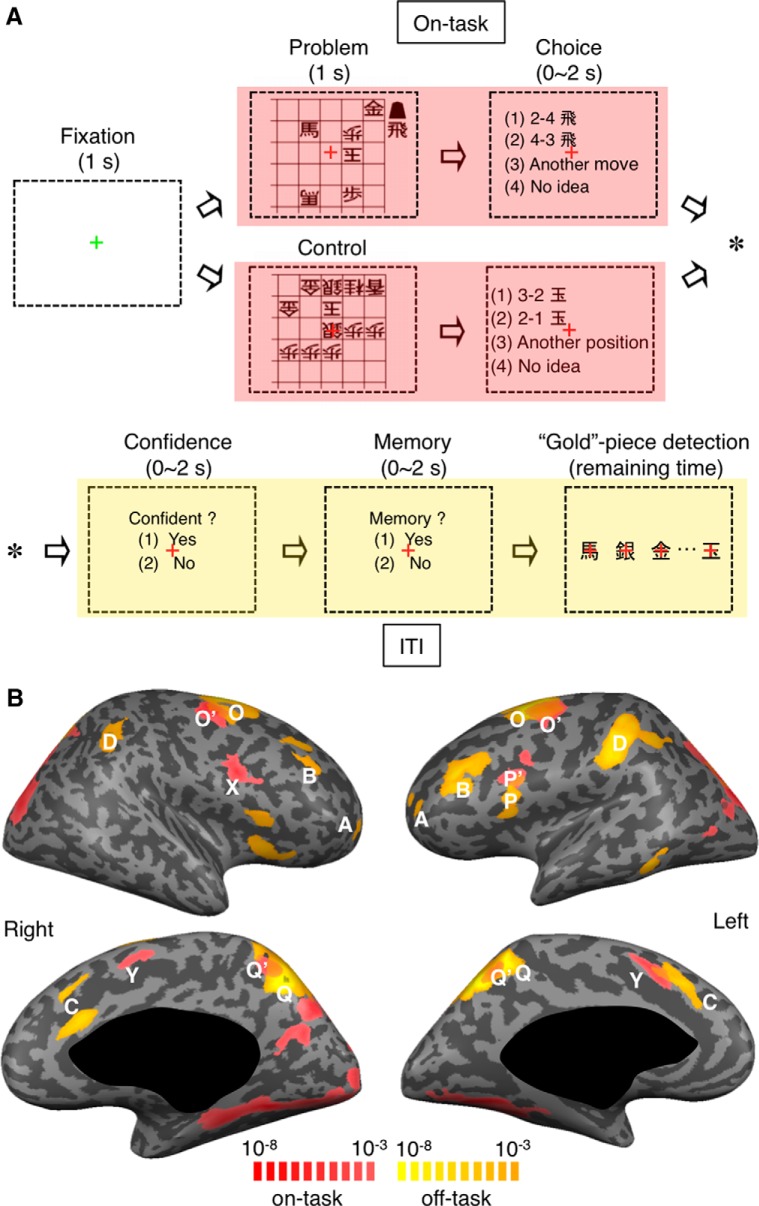
Tasks and activation patterns in Experiment 1. ***A***, Sequence of main task events in each trial. Red and yellow shadings represent the on-task and ITI periods, respectively. ***B***, The on-task activation (red) determined by comparing BOLD signal changes during the generation task with those during the ITI period after the generation task, and the off-task activation (yellow) determined by comparing signal changes during the ITI period after the generation task with those during the ITI period after the control task. The results shown in Figures 1*B*, 2, and 3 were obtained using half of the data from Experiment 1. A, lFPC; B, mDLPFC; C, dACC; D, aIPL; X, pDLPFC (right); Y, pre-SMA; O and O’, PMd; P and P’, pDLPFC (left); Q and Q’, precuneus.

***Experiment 1.*** Trials of quick generation task were intermingled with those of sensory-motor control task (see Fig. 1*A*). The subject was first presented with a board pattern in both tasks. In the generation task, the board pattern provided a checkmate problem, for which the subject generated the idea of the first move of the move sequences that would reach the final checkmate (capturing the opponent’s king) against the optimum counter moves of the opponent. In the control task, the board pattern was composed only of opponent’s pieces, among which the subject had to find the king piece. As there were no pieces of the subject’s side, the subject could not think about the next-move. For both next-move generation and control tasks, after selecting the answer from four options, the trial proceeded to an intertrial interval (ITI) period, during which the subject answered two simple questionnaires and then performed a distractor task.

In detail, each trial started with the appearance of a fixation point. After a 1 s fixation, the board pattern of a checkmate problem (in the generation task) or that composed only of the opponent’s pieces (in the control task) appeared for 1 s. Four choice options were then presented, and the subject selected the one that matched his idea of the first move (in the generation task) or of the king’s position (in the control task) within 2 s. While general checkmate problems require reports of the sequence of moves that would reach the final checkmate together with the optimum counter moves of the opponent, we asked our subjects just to report their ideas of the first move of the sequence.

In the questionnaires given at the beginning of the ITI period, the subject reported his confidence in the previous choice and then whether he made the choice by recalling the memory of the problem (see the next paragraph). In the following distractor task during ITI, the subject was presented with shogi pieces one at a time at a rate of four pieces per second (150 ms each followed by a mask for 100 ms) and reported the appearance of a “Gold” piece by pressing a button. We intended to stop the thinking of the previous problem by the distractor task. The total duration of a trial was fixed to 11 s. Because the trial proceeded to the next phase at the subject’s button press in the main task and questionnaires, the period of the distractor task varied from 3 to 8 s.

We gave 180 trials of the generation task and 60 trials of the sensory-motor control task to each subject. The board pattern was trial unique. The checkmate problems were newly created by professional players belonging to the Japan Shogi Association. Their difficulty varied with 7-15 moves required to reach the final checkmate, including the opponent’s counter moves, so that they were challenging, and not too difficult, for both professional and high-level amateur players. As the checkmate problems were newly created, the subject rarely reported the problems’ memory in the second questionnaire (8% for the professional players and 3% for the amateur players). Some more details of the task have been described previously (Wan et al., 2011).

***Experiment 2.***To compare brain activities associated with quick generation of the best next-move with those associated with deliberative search of the best next-move, we conducted Experiment 2 (with deliberative search) following Experiment 1 (with quick generation) in each fMRI session. We randomly selected 30 checkmate problems from the problems that the subject failed to give correct answers in Experiment 1. Each problem was presented for up to 8 s, during which the subject was instructed to search deliberately to find the best next-move (see Fig. 5*A*). When the subject pressed a button within 8 s, the trial proceeded to the answer-selection phase. Otherwise, the task entered automatically to the answer selection phase at the end of 8 s. After the subject chose the answer from the four options within 2 s, the trial proceeded to the ITI period occupied with the distractor task (the “Gold” piece detection). Unlike Experiment 1, there was no questionnaire for confidence or memory report or sensory-motor control trial. The length of each trial was fixed to 16 s. The period of ITI varied more (between 5 and 13 s) than the variation in Experiment 1.

***Experiment 3.*** The basic structure of the task paradigm was the same as that in Experiment 1, but to further examine properties of the post-task activations found in Experiments 1 and 2, three conditions were introduced during ITI. After the quick next-move generation task, the subject was engaged in (1) the “Gold” piece detection, which was used in Experiments 1 and 2; (2) fixation only (“rest” condition); or (3) rethinking the preceding problem (“rethink” condition) (see Fig. 8*A*). After the sensory-motor control trials, only the first two conditions (“Gold” piece detection and “rest” conditions) were provided. The task sequence was similar to that used in Experiment 1. After a 0.5 s fixation period, a checkmate problem or a board pattern for the detection of the king was presented for 2 s, and the subject was then instructed to choose one from four options within 2 s. Unlike Experiments 1 and 2, the screen for selection remained until the end of the 2 s period, even after the subject had pressed the button. Subsequently, an instruction indicating the condition during the upcoming ITI was briefly shown for 0.5 s, followed by ITI for 4 s. At the end of the ITI period, the subject either chose, within 2 s, his answer again from four options in the “rethink” condition or simply pressed the button marked in red in the other two conditions. The length of each trial was 11 s, and there was no questionnaire for confidence or memory report. There were 60 trials for each combination of on-tasks and ITI conditions, and a total of 300 trials were given to each subject. The order of the five types of trials was random, except that a control trial was always followed by a generation trial. Of 180 generation trials, 120 were preceded by a control trial and 60 were preceded by a generation trial.

***Experiment 4.*** The subjects who had had no prior experience of shogi were daily trained for playing games of a simple shogi (“gogo” shogi) for 15 weeks, and the fMRI experiments were conducted twice on each subject: at the early (the 2-3 weeks) and end (the 14-15 weeks) phases of the training. The subject practiced the exercise, on average, 40 min per day. Gogo-shogi uses a 5 × 5 board, in place of a 9 × 9 board in original shogi, and fewer types of pieces. A game of gogo-shogi is completed with fewer moves (typically ∼30, including both sides) than moves for a typical game of original shogi (∼120). The basic structure of the task paradigm used in the fMRI experiments was the same as that in Experiment 1: trials of the correct next-move generation task were intermingled with those of the sensory-motor control task, and the main task was followed by the ITI period occupied with the distractor task (“Gold” piece detection). Unlike Experiment 1, however, checkmate problems of gogo-shogi were used for the correct next-move generation task, the board pattern was presented for 2 s, the subject chose the answer within 3 s, all the four choice options were concrete moves or positions, and there were no questionnaires. The length of each trial was 11 s, and 180 next-move generation trials together with 60 sensory-motor control trials were given to each subject, as in Experiment 1. The next-move generation trials were randomly intermingled with the control trials.

### MRI specifications

All fMRI experiments were conducted using a 4 T MRI system with a head gradient coil (Agilent). A birdcage radiofrequency transmit-receive coil was used in Experiments 1 and 2. A combination of a birdcage radiofrequency transmit coil and 4 phased-array receive surface coils was used in Experiments 3 and 4.

***Experiments 1 and 2.*** Functional images were acquired using a two-segment center-ordered gradient echo T_2_^∗^ EPI sequence with volume TR of 2 s, TE of 15 ms, slice thickness of 5.5 mm, and in-plane resolution of 3.75 × 3.75 mm^2^ (FOV: 24 × 24 cm^2^; flip angle: 40 degrees). Twenty-one axial slices, parallel to the anterior commissure-posterior commissure line, were acquired with an interleaved acquisition procedure.

***Experiments 3 and 4.*** Functional images were acquired using a TSENSE technique and a two-segment center-ordered gradient echo T_2_^∗^ EPI sequence with volume TR of 2 s, TE of 15 ms, slice thickness of 4.0 mm, and in-plane resolution of 3.0 × 3.0 mm^2^ (FOV: 19.2 × 19.2 cm^2^; flip angle: 40 degrees). Thirty oblique slices, oriented 15 degrees from the anterior commissure-posterior commissure line, were collected with an interleaved acquisition procedure.

### fMRI data analyses

***Preprocessing.***After reconstruction of EPI images, data analyses were performed using BrainVoyager (Brain Innovation). To correct for the rigid head motion, all EPI images were realigned to the first volume of the first scan. Datasets in which translation motions were >1.0 mm or rotation motions were >1.0 degree were discarded. Functional EPI images were then transformed into the Talairach space by resampling the data with a resolution of 2 × 2 × 2 mm^3^. A spatial smoothing with a 4 mm Gaussian kernel (FWHM) and a high-pass temporal filtering with a cutoff of 0.005 Hz were applied to all fMRI data.

***Determination of post-task period and associated regions of interest (ROIs).*** GLM regression analyses were used to determine ROIs of activation and for several other analyses. All regression analyses used two regressors: one obtained by convolving the on-task period with a canonical hemodynamic function (HRF) and the other by convolving the ITI period or post-task period with the canonical HRF.

The on-task period started at the onset of the problem presentation; and in Experiments 1, 3, and 4, it included the entire time for problem presentation and the difference in response time, obtained by subtracting the mean response time of the subject in the sensory-motor control task from the response time in individual trials. The mean response time in the control task was subtracted from the response time in individual trials because the former time was assumed to be used for perceiving the options and pushing a button. The remaining time was likely used for thinking the problem. For Experiment 2, the on-task period was fixed to the problem presentation time. As the subjects voluntarily terminated the problem presentation to move on to the option selection, we assumed that they did not continue to think the problem after the termination of the problem.

The ITI-period regressor was used only for the initial analysis of the activation in Experiment 1. The ITI period started at the time when the subject made the choice, which initiated two questionnaires, and covered the periods for the questionnaires and for the distractor task (“Gold” piece detection). Half of the data in Experiment 1 was used to determine the activation (see Fig. 1*B*); and then, by deconvolving the mean time course of the obtained BOLD signal change in each ROI for this off-task activation, the onset and duration of the neural activation that evoked BOLD signal changes were estimated (see Fig. 3). The initial and end positions were averaged among the four ROIs to determine a common window for analyses of the post-task activation (post-task window). The estimated initial and end positions of the post-task were 0.3 and 3.8 s, respectively, after the onset of the first questionnaire. A regressor obtained by convolving this post-task activation period with the canonical HRF was used for the second analysis of the activation in Experiment 1 and for Experiments 2-4. The second analysis of the activation in Experiment 1 used the remaining half of the data to redefine the ROIs for on-task and post-task activations (see Fig. 4*A*). These ROIs were subsequently used for all ROI analyses presented in this paper. For Experiments 2 and 4, we assumed that the post-task period started at the time when the subject made the choice, which initiated the distractor task in these experiments. For Experiment 3, we assumed that the post-task period started at the end of instruction for the ITI task (0.5 s after the subject made the choice).

Each of the on-task-period, ITI-period, and post-task-period regressors was differentiated into two: one for trials of quick next-move generation task and the other for trials of sensory-motor control task. For Experiment 3, each of the two post-task-period regressors was further differentiated into three according to the ITI conditions. The variation in response time across trials was taken into consideration in the regression analysis, as the length of the on-task period was changed depending the differences in the response time.

To determine activated voxels, regression beta coefficients calculated for each individual subject were used for a group random-effect ANOVA. For Experiments 1 and 2, in which both amateur and professional players participated, after a group random-effect analysis within each subject group, a conjunction analysis across subject groups was also performed. Multiple comparison corrections were performed by calculating the false discovery rate (FDR, *p* < 0.05 after correction) throughout the whole brain. Unless noted otherwise, ROI analyses were based on data from both hemispheres.

***Analysis of functional connectivity between the on-task and post-task networks.*** The correlation of trial-by-trial variances in activities was examined by using the data obtained in Experiment 1 as follows (see Fig. 10). (1) The response at each time was averaged across voxels within each ROI of each subject. (2) The mean response was integrated over time with weights of the function obtained by convolving the canonical HRF with the on-task period (for the on-task network) or post-task period (for the post-task network). (3) Deviations of the trial-by-trial responses from the mean averaged over all the trials were calculated. (4) The coefficient was calculated for the correlation of the deviations for each pair of ROIs in the on-task and post-task networks (one ROI from the on-task network and the other ROI from the post-task network). (5) The coefficient was averaged over all the ROI pairs. (6) The mean coefficients in individual subjects of each subject group (professional or amateur) were converted by Fisher’s *z*-transformation. (7) The significance of the correlation between post-task activation and preceding on-task activation, between post-task activation and succeeding on-task activation, and between successive on-task activations was statistically examined by applying a two-tailed, one-sample *t* test to the distributions of converted coefficients across subjects within each subject group. As individual time points of BOLD signals in each trial are not independent, the degrees of freedom were modified by the Bartlett correction factor.

**Figure 2.**
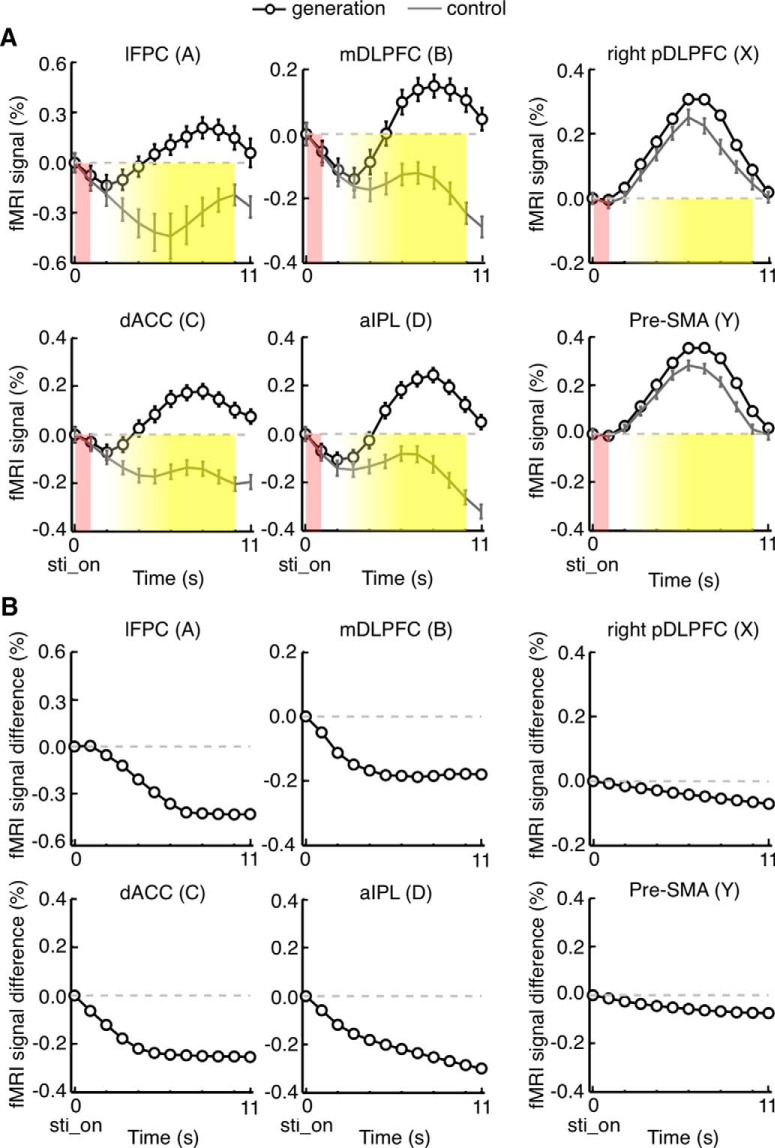
Initial decreasing trends in BOLD signal changes that commonly occurred in generation and control trials. BOLD signal changes were aligned to the onset of problem presentation (sti_on). ***A***, The original BOLD signal changes in the generation trials preceded by a generation trial (black lines) and those in the control trials preceded by a generation trial (gray lines), respectively, in Experiment 1. Error bars indicate SEM across subjects. Red shadows represent the board position presentation period. Yellow shadows represent the ITI period. Mean signal changes were vertically shifted so that the value at time zero became zero. ***B***, The initial decreasing trends in Experiment 1, extracted by calculating BOLD signal differences between the generation trials preceded by a control trial and the generation trials preceded by a generation trial.

***Adjusting time courses of BOLD signal changes in Experiments 1 and 3.***The ITI periods used in the present study were not long enough for the BOLD signal to completely return to the baseline level within each trial. To remove the general initial declining trends of BOLD signal changes caused by neural activations in the previous generation-task trial (for Experiment 1, see Fig. 2*A*), we calculated differences in the mean time course between generation-task trials preceded by a generation-task trial (g-G trials) and generation-task trials preceded by a control-task trial (c-G trials) (for Experiment 1, see Fig. 2*B*) and subtracted the mean differential time course (g-G – g-C trials) from the time courses in individual trials preceded by a generation-task trial (g-G and g-C trials). The declining trends were estimated separately for Experiments 1 and 3. As there were no control-task trials in Experiment 2, original response time courses were used for Experiment 2 (see Figs. 6,7).

***Fitting of fMRI signal change time courses in Experiment 2.*** We used the data obtained in Experiment 2 (deliberate search) to determine the task event to which neural activations that caused BOLD signal changes were locked. We divided the trials into three groups according to the subject’s responses: the trials that the subject terminated within 4 s (2.8 ± 0.3 s, mean ± SE), which are referred to as quick-search trials; those that the subject terminated between 4 and 8 s (6.3 ± 0.3 s, slow-search trials); and those during which the subject did not press the button to terminate. We did not include the last group of trials in the main analysis because the subjects likely continued to think the given problem in these trials even after the problem presentation was already terminated. We determined a model that consistently explained the time courses of BOLD signal changes in quick-search and slow-search trials by using the following formulas:

(1) $${BOLD}_{fit}=a\;*\;conv(HRF,Na)+b$$

where

(2) $$HRF=\frac{{\left(\frac{t}{\tau }\right)}^{n-1}e^{-\frac{t}{\tau }}}{\tau \Gamma \left(n-1\right)}$$

and *a* and *b* are the linear parameters to adjust the magnitude and bias of BOLD signal changes. *conv* represents the convolution, *HRF* a hemodynamic response function, *n* a parameter to adjust the shape of *HRF*, and τ a parameter to adjust the width of *HRF*. *Na* represents the position and duration of neural activation that evoked BOLD signal changes. We examined the performance of two models. Model 1 assumed that *Na* started at the problem onset and lasted for the problem presentation period, which varied across trials. Model 2 assumed that *Na* started at the time when the subject made the choice (i.e., the ITI onset) and lasted for 3.5 s (see above). Because there were no sensory-motor control trials in Experiment 2, we used original BOLD signals. Although the deconvolution method was used to reduce the contamination of responses from the preceding trial, it was only partly successful: a decreasing phase of BOLD response from the previous trial remained (see Fig. 6). The mean time courses aligned to the onset of the problem presentation were fitted with Model 1 (see Fig. 6*A*), and those aligned to the subject’s option selection (i.e., the ITI onset) were fitted with Model 2 (see Fig. 6*B*). The Levenberg-Marquardt optimization algorithm (“fminsearch” in MATLAB 7.7, The MathWorks) was used to determine the set of *a*, *b*, *n*, and τ that gave the best fit for each model. Finally, the mean square error (MSE) was compared between the two models (see Table 2).

## Results

We used the checkmate problem in the game of shogi in the present study. The checkmate problem is a well-designed puzzle, a type of rule-based problem-solving task. For each problem (or board position), there exists only one correct solution, which is a sequence of moves that reaches the final checkmate (capture of the opponent’s king), even with optimal counter moves by the opponent. The number of moves required before reaching the final checkmate varied from 7 to 15 (including the opponent’s moves). Although checkmate problems usually require the player to report the entire sequence of moves that reaches the final checkmate, we asked our subjects just to report their ideas of the first move, so as to emphasize the rapid generation of the best next-move. Players with high proficient levels can quickly generate, for most of problems, an idea of the first move of the sequence that may reach the final checkmate. Experiments 1 and 2 were originally designed to identify neural substrates of the process in expert players to quickly generate the best next-move (Wan et al., 2011). Experiment 4 was designed as a follow-up study to examine the development of the capability along a long-term training and of associated brain activities in subjects who had been novices before the training (Wan et al., 2012). The present paper mainly reports the unexpected brain activities that occurred after the subject completed the decision. Experiment 3 was newly designed to further examine properties of these brain activities.

### fMRI responses during the off-task or post-task period

In Experiment 1, trials of quick next-move generation task (to report the best next-move to the given checkmate problem) were intermingled with those of sensory-motor control task (to report the position of the “King” piece in the given board, which was exclusively composed of the opponent’s pieces) (Fig. 1*A*; see Experiment 1). In both generation and control trials, after the subject selected the answer, the trial proceeded to the ITI period that was occupied with two simple questionnaires and a distractor task (to detect the appearance of “Gold” piece in sequentially presented pieces). Although the length of a trial was fixed to 11 s, because the times used by the subject for making the choice and answering the two questionnaires in each trial varied (1.26 ± 0.35 s and 1.31 ± 0.59 s, respectively, mean ± SD across trials), the length of the distractor task also varied across trials (3-8 s, 6.41 ± 0.75 s, mean ± SD across trials). BOLD signal changes were analyzed with two regressors: (1) the on-task (i.e., online processing) period covering the problem presentation (1 s) and the response times in individual trials subtracted by the mean response time of the subject in the sensory-motor control task (0.40 ± 0.33 s, mean ± SD across trials), convolved with the canonical HRF; and (2) the ITI period occupied with the questionnaires and distractor task, convolved with the canonical HRF.

**Figure 3.**
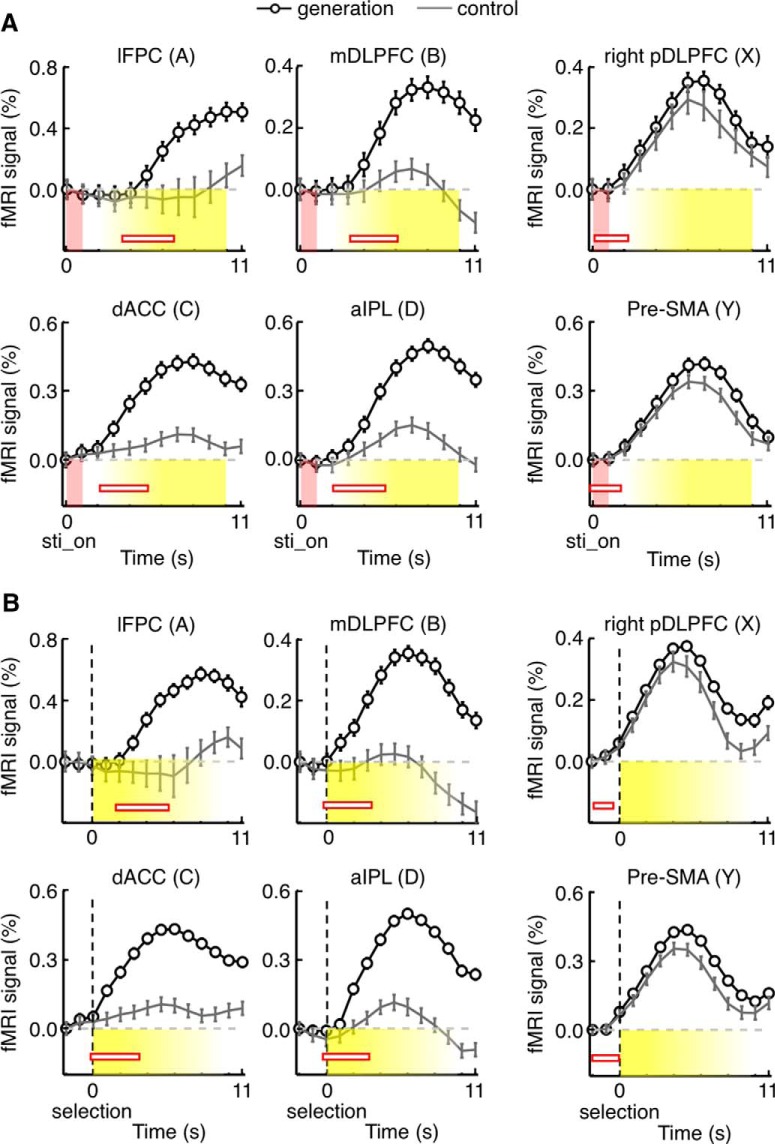
Time courses of on-task and post-task (off-task) activations in Experiment 1. ***A*,** BOLD signal changes aligned to the onset of problem presentation (sti_on). Mean signal changes were vertically shifted so that the value at time zero became zero. The effects from the previous generation trial were removed by subtracting the differences between mean signals in generation trials preceded by a generation trial and those in generation trials preceded by a control trial (shown in Fig. 2*B*) from the time courses of signal changes in individual trials. Other conventions are the same as in Figure 2*A*. Horizontal red open bars represent the position and duration of neural activations determined by deconvolving mean BOLD signal changes in generation trials with a canonical HRF. ***B***, The same as in ***A***, but BOLD signal changes were aligned to the subject’s option selection (selection), which was also the onset of questionnaires. Mean signal changes were vertically shifted so that the value at 2 s before time zero became zero.

Several cortical areas, including the pDLPFC (or inferior frontal junction; BA 8/9), pre-SMA, dorsal premotor cortex (PMd, BA 6), and posterior precuneus (BA 7), were activated during the on-task period of the next-move generation task compared with the off-task period after the next-move generation task (*p* < 0.05, FDR corrected; Fig. 1*B*, red patches, Figs. 2,3, right; Table 1), as we had reported previously (Wan et al., 2011). Activations in these areas during the sensory-motor task were as strong as those during the next-move generation task in Experiment 1 (Figs. 2,3, right), whereas their activations during the sensory-motor task were much smaller than those during the next-move generation task in Experiment 3 (see Fig. 9, right), where the board positions were presented for a longer period (2 s) and the proportion of control trials was higher. Cortical areas in the rostral frontal cortex, including FPC, mDLPFC, and dACC, which have been repeatedly reported to be associated with cognitive control, were not activated during online processing of quick next-move generation (Figs. 2,3, left).

**Table 1.** The brain areas in which fMRI activities were higher during the on-task period compared with those during the post-task period in the quick next-move generation task (on-task activation), and the brain areas in which fMRI activities were higher during the post-task period after the quick next-move generation task compared with those during the post-task period after the sensory-motor control task (post-task activation) in Experiment 1 (*p* < 0.05, FDR corrected)

Brain region		Talairach coordinates			No. of voxels
		*x*	*y*	*z*	
**Post-task activation**					
lFPC	L	-27	53	11	123
	R	23	55	10	43
mDLPFC	L	-40	29	33	214
	R	35	31	33	125
dACC*^a^*		-2	24	44	79
Left pDLPFC	L	-44	8	20	97
PMd	L	-22	2	54	422
	R	20	2	58	296
aIPL	L	-51	-29	44	943
	R	35	-35	39	268
Precuneus	L	-8	-64	48	452
	R	4	-60	48	496
**On-task activation**					
pDLPFC	L	-43	6	28	104
	R	39	5	27	92
Pre-SMA	L	-7	8	47	114
	R	5	7	48	45
PMd	L	-22	-7	51	216
	R	24	-10	53	228
Precuneus	L	-16	-60	44	162
	R	15	-59	47	176

*^a^*This cluster included voxels in both hemispheres.

**Table 2.** The parameters that provided the optimal fitting of the mean fMRI time courses in each ROI obtained from Experiment 2 with two models*^a^*

					Quick search		Slow search		
	Subject group	Model	*n*	τ	*a*	*b*	*a*	*b*	MSE
**On-task network**									
Right pDLPFC	All	1	5	1.52	1.07	0.03	0.85	-0.01	0.20
		2	4	0.83	0.48	-0.12	0.72	-0.13	0.30
Pre-SMA	All	1	5	1.39	1.32	0.00	0.99	-0.07	0.15
		2	4	0.70	0.70	-0.12	0.88	-0.17	0.44
**Post-task network**									
lFPC	All	1	7	1.32	0.82	-0.20	0.45	-0.24	0.40
		2	4	1.63	1.03	-0.05	1.25	-0.04	0.07
	1	1	7	1.38	0.79	-0.23	0.58	-0.18	0.42
		2	4	1.67	1.02	-0.08	1.28	-0.01	0.09
	2	1	7	1.36	0.84	-0.18	0.54	-0.20	0.44
		2	4	1.68	1.05	-0.07	1.26	-0.02	0.11
mDLPFC	All	1	7	1.25	1.17	-0.26	0.72	-0.27	0.79
		2	4	1.26	0.86	0.04	1.17	-0.03	0.22
	1	1	7	1.30	1.09	-0.22	0.94	-0.23	0.74
		2	4	1.29	0.90	0.01	1.21	0.02	0.24
	2	1	7	1.32	1.22	-0.19	1.06	-0.21	0.85
		2	4	1.31	095	0.03	1.24	0.04	0.26
dACC	All	1	7	1.04	1.50	-0.08	0.89	-0.22	0.34
		2	5	0.82	1.15	0.01	1.23	-0.09	0.14
	1	1	7	1.08	1.03	0.04	1.12	-0.28	0.39
		2	5	0.88	1.12	0.08	1.25	-0.04	0.16
	2	1	7	1.25	1.42	-0.06	0.94	-0.23	0.43
		2	5	0.90	1.18	0.05	1.21	-0.02	0.19
aIPL	All	1	7	1.28	1.64	-0.23	0.82	-0.33	0.54
		2	4	1.34	1.27	-0.04	1.32	-0.14	0.24
	1	1	7	1.35	1.58	-0.28	0.89	-0.37	0.59
		2	4	1.36	1.29	-0.02	1.28	-0.10	0.29
	2	1	7	1.32	1.62	-0.26	0.88	-0.32	0.56
		2	4	1.32	1.32	-0.06	1.29	-0.07	0.26

*^a^*MSE indicates the goodness of the fitting. The 32 subjects who participated in Experiment 2 were divided into two groups: Group 1, with more quick-search trials than slow-search trials (*n* = 16); and Group 2, with more slow-search trials than quick-search trials (*n* = 16).

In contrast, when we compared activities during the ITI period after the next-move generation task with those during the ITI period after the sensory-motor control task, widespread cortical areas were activated (*p* < 0.05, FDR corrected, Fig. 1*B*, yellow patches, and Figs. 2,3, left), although the subjects were engaged in the identical “Gold” piece detection task in both conditions. The areas that exhibited such an off-task activation comprised lFPC (BA 10), mDLPFC (BA 9/46), dACC (BA 8/32), left pDLPFC, PMd, and aIPL (BA 5/7), and the posterior precuneus (Table 1).

**Figure 4.**
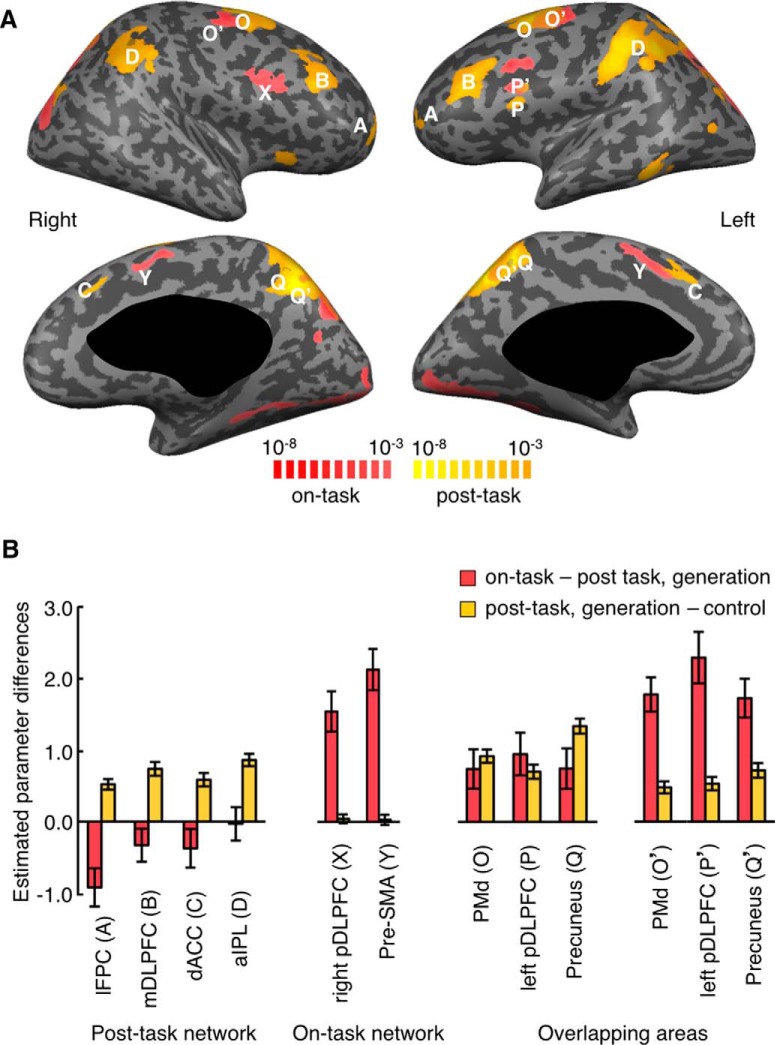
Recalculated activation patterns and selectivity in Experiment 1. ***A***, Similar to those in Figure 1*B*, but obtained by using the second half of the data from Experiment 1 and the post-task period. Conventions are the same as in Figure 1*B*. ***B***, Magnitudes of on-task and post-task activations in the post-task network areas, on-task network areas, and overlapping areas, obtained by using the second half of the data from Experiment 1. Red bars represent differences between β values for the on-task period regressor in generation-task trials and those for the post-task period regressor in generation-task trials. Yellow bars represent differences between β values for the post-task period regressor in generation-task trials and those for the post-task period regressor in control-task trials. Error bars indicate SEM across subjects.

In some areas, the region that showed off-task activations after the next-move generation task partially overlapped with the region activated during the online processing of the next-move generation task (left pDLPFC, bilateral PMd, and bilateral posterior precuneus) (Fig. 1*B*). The selectivity for the on-task or off-task activation was weaker in these regions (Fig. 4*B*), as can be expected from the partial overlapping between on-task and off-task activation regions. To further examine the properties of the on-task and off-task activation, we focused on the regions that showed only the on-task or off-task activation. We refer to the regions activated only during online processing of next-move generation (right pDLPFC and bilateral pre-SMA) as the on-task network, and the regions activated only during the ITI period after the quick next-move generation (bilateral lFPC, bilateral mDLPFC, dACC, and bilateral aIPL) as the off-task network. The off-task network largely coincided with the “frontoparietal control network” (Vincent et al., 2008), but not with the “default-mode network” activated during rest (Buckner et al., 2008).

The mean time courses of BOLD signal changes are shown in Figure 2*A*. Among the 180 trials of the next-move generation task, we focused on the 120 trials that were preceded by a next-move generation trial. Activities in these generation trials were contrasted with those in the 60 trials of the sensory-motor control task, which were all preceded by a next-move generation trial. There were initial decreasing trends commonly in both groups of trials (Fig. 2*A*), which likely represented the late part of signal changes caused by neural activations in the preceding generation trial. They were estimated by taking the differences between the mean signals in generation trials preceded by a generation trial and those in generation trials preceded by a control trial (Fig. 2*B*). The rectified mean time courses of BOLD signals obtained by subtracting the estimated initial decreasing trends from signal changes in individual trials are shown in Figure 3. Signal changes in individual trials were aligned to the beginning of the on-task period (i.e., the onset of problem presentation) in Figure 3*A*, as in Figure 2, and to the beginning of the ITI period (i.e., the onset of questionnaire period) in Figure 3*B*. In the regions of the off-task network, the BOLD signals in generation trials (black traces) started to increase and deviate from those in control trials (gray traces) 3-5 s after the task onset, and the signals in generation trials peaked toward the end of the trial. The time difference between this peak in BOLD signals in the off-task network (on average, 9 s after the onset of the board position presentation) and the peak in the BOLD signals in the on-task network (on average, 6 s after the onset of the board position presentation) was ∼3 s, which largely matched the mean time difference between the onset of the board position presentation and the onset of the ITI period (3.54 ± 0.01 s, mean ± SEM across subjects).

By deconvolving these BOLD signals with the canonical HRF, we estimated the position and duration of the neural activations that led to BOLD signal changes in the generation trials (Fig. 3*A*,*B*, red horizontal bars). Whereas neural-activation periods estimated for the on-task network matched the on-task period relatively well, those estimated for the off-task network started approximately at the beginning of the ITI period. However, estimated neural-activation periods in the off-task network were much shorter than the off-task period: they all ended in the middle of the ITI period. Thus, we decided to use the estimated period of neural activation, averaged across lFPC, dACC, mDLPFC, and aIPL, as the second regressor. This period started 0.3 s after the subject’s option selection, or the onset of questionnaires, and ended 3.8 s after the subject’s option selection. This 3.5 s period was termed the “post-task period.” Statistical activation maps were generated again using one regressor associated with this post-task period and the other with the on-task period (Fig. 4*A*). To avoid circularity of analysis, the initial determination of activated voxels and the estimation of position and period of neural activations that led to BOLD signal changes were made using a half of the data (odd runs), and activated voxels were determined again with the estimated neural-activation period using the remaining half of the data (even runs). Although activation maps calculated by the post-task-period regressor were almost identical to those calculated by the off-task-period regressor, we hereafter refer to the activation determined by the post-task-period regressor as the “post-task activation” and to the network of regions that showed significant post-task activation as the “post-task network,” for clarity.

### Late-onset BOLD signal changes can be explained by late neural activations, but not by slow hemodynamic responses

**Figure 5.**
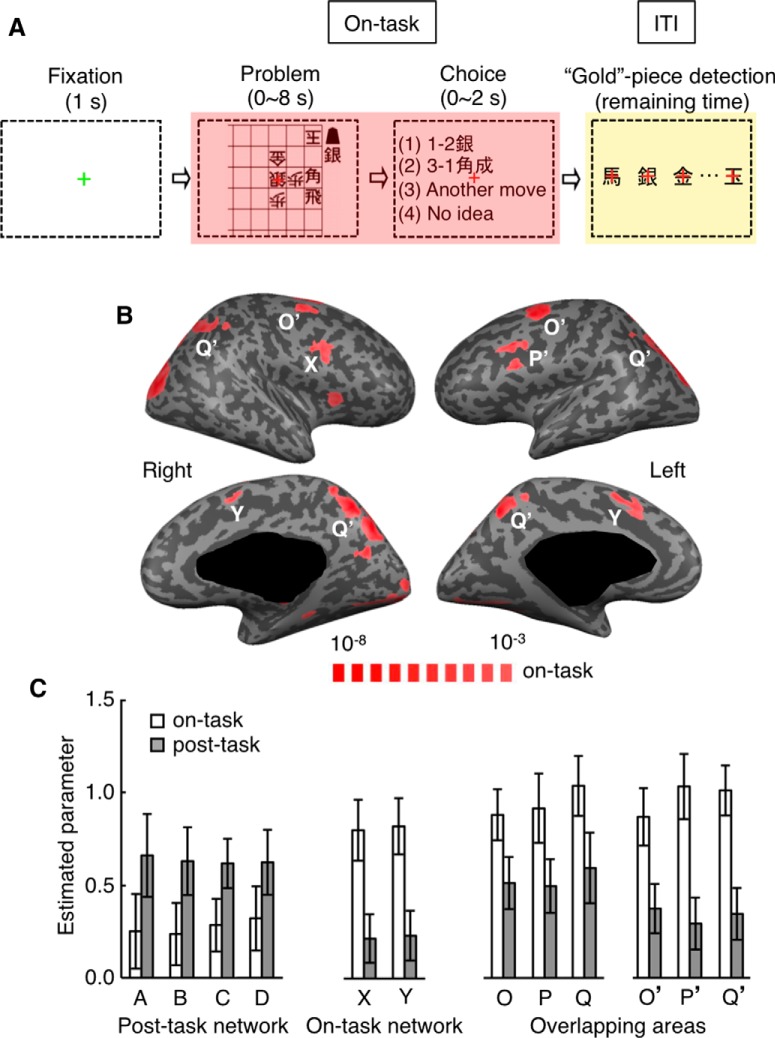
Tasks and activation pattern in Experiment 2. ***A***, Sequence of main task events in each trial. ***B***, Statistical parametric maps for the on-task activation (the next-move generation period contrasted with the post-task period). ***C***, β values determined by GLM analyses for the on-task and post-task period regressors. Error bars indicate SEM across subjects.

In Experiment 2, the subjects were given longer time (8 s) to search for the best next-move while they were allowed to move to answer anytime within 8 s by pressing a button (Fig. 5*A*; see Experiment 2). The time was long enough for the subjects, who were proficient in shogi, to make deliberative search. Similar on-task and post-task activation patterns as those in Experiment 1 were observed in Experiment 2 (Fig. 5*B*,*C*). The presence of post-task activations after deliberative search indicated that the post-task activations occurred commonly after the quick generation and deliberative search of the best next-move to a given checkmate problem.

Experiment 2 also provided a good opportunity to confirm that the post-task activations in the post-task network were caused by neural activations occurring after the completion of the decision in the next-move generation task, as the trial-by-trial variance of the interval between the onset of board position presentation and beginning of the ITI period was larger in Experiment 2 (SD, 3.6 s) than that in Experiment 1 (0.4 s). We focused on two groups of trials selected based on the task duration: the trials in which the subject moved to answer within 4 s (quick-search trials), and those in which the subject moved to answer between 4 and 8 s (slow-search trials). Trials in which the subjects completed the 8 s period without pressing the button were not included in the analyses below because the way by which the subject finished the search in these trials was different from that in the other trials (passive vs active). The numbers of quick- and slow-search trials were equal (25% and 25%), and the mean search durations in quick- and slow-search trials were 2.8 and 6.3 s, respectively.

**Figure 6.**
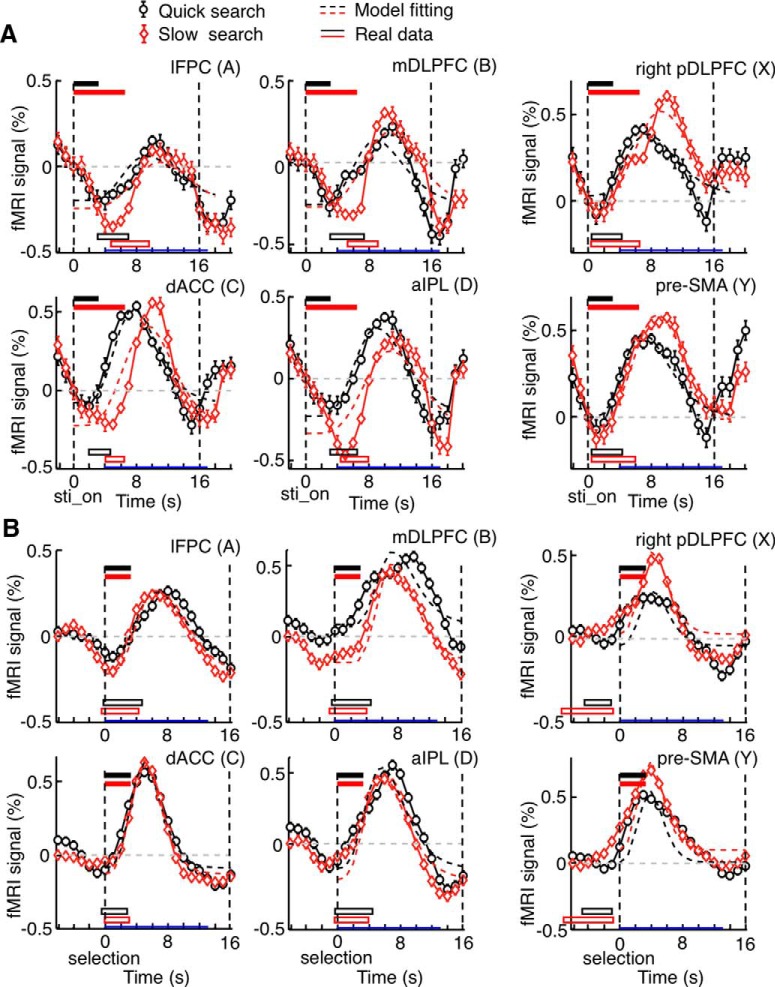
Activation time courses in Experiment 2. ***A***, Mean BOLD signal changes aligned to the onset of the problem presentation (sti_on), obtained by the deconvolution method for the trials with problem presentation shorter than 4 s (on average, 2.8 s, quick-search trials, solid black lines), and those obtained for the trials with problem presentation between 4 and 8 s (on average, 6.3 s, slow-search trials, solid red lines). Horizontal black and red filled bars represent mean problem-presentation periods, in quick-search and slow-search trials, respectively. ***B***, Mean BOLD signal changes aligned to the subject’s option selection (selection), which was also the onset of the ITI period. Horizontal black and red filled bars represent the post-task period, starting at the onset of the ITI onset. ***A***, ***B***, Mean signal changes were vertically shifted so that the value at the problem-presentation onset was zero. Error bars indicate SEM across subjects. Horizontal open black and red bars represent the estimated neural activation period in quick-search and slow-search trials, respectively. Broken black and red lines indicate the time courses determined by optimally fitting HRF parameters with the neural activation coincided, presumably, with the on-task period in ***A*** or the post-task period in ***B***. Blue line segments overlaid on each horizontal axis indicate the period during which the MSE was calculated for the fitting.

BOLD signals were aligned to the onset of the search period in Figure 6*A* and to the option selection (i.e., ITI onset) in Figure 6*B*, for quick-search trials (black traces) and slow-search trials (red traces). To examine whether late-onset BOLD signal changes in the post-task network were caused by neural activations during the generation task or those after the termination of the task, we adjusted shape parameters of the HRF (a gamma function) to fit BOLD signal changes: the HRF was convolved with the period of generation task in Model 1 and with the 3.5-s off-task period, starting at the option selection, in Model 2 (see Fitting of fMRI signal change time courses in Experiment 2). Shape parameters of the HRF in each area were adjusted separately in the two models but common to quick-search and slow-search trials. Figure 6*A*, *B* (dashed traces) indicates the time courses of the models that fitted the data optimally. Model 2 fitted BOLD signal changes better in all the regions of the post-task network, whereas Model 1 fitted BOLD signal changes better in all the regions of the on-task network (Table 2).

**Figure 7.**
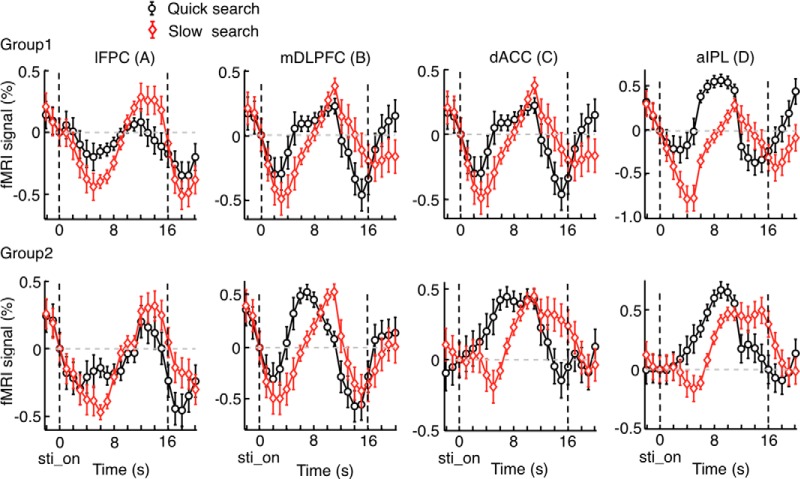
Activation time courses in two groups of subjects in Experiment 2: Group 1 in which the quick-search trials dominated and Group 2 in which the slow-search trials dominated. BOLD signal changes were aligned to the onset of the problem presentation. Conventions are the same as Figure 6*A*. The time course of the slow-search trials (red lines) was delayed compared with that of the quick trials (black lines) in each region commonly in both subject groups.

The ratio of the number of quick-search trials to that of slow-search trials varied across subjects. Thus, it was possible that the differences in BOLD time courses between quick- and slow-search trials, as shown in Figure 6, merely reflected the differences between subjects, but not between trials. To rule out this possibility, we divided the subjects who participated in Experiment 2 into two groups: one with more quick- than slow-search trials and the other with more slow-search trials. We found that the time courses of BOLD signal changes in the post-task network were similar between the two groups of subjects (Fig. 7) and that Model 2 consistently better fitted BOLD signal changes in the post-task network in both groups of subjects (Table 2).

### Robust post-task activation regardless of conditions during ITI

**Figure 8.**
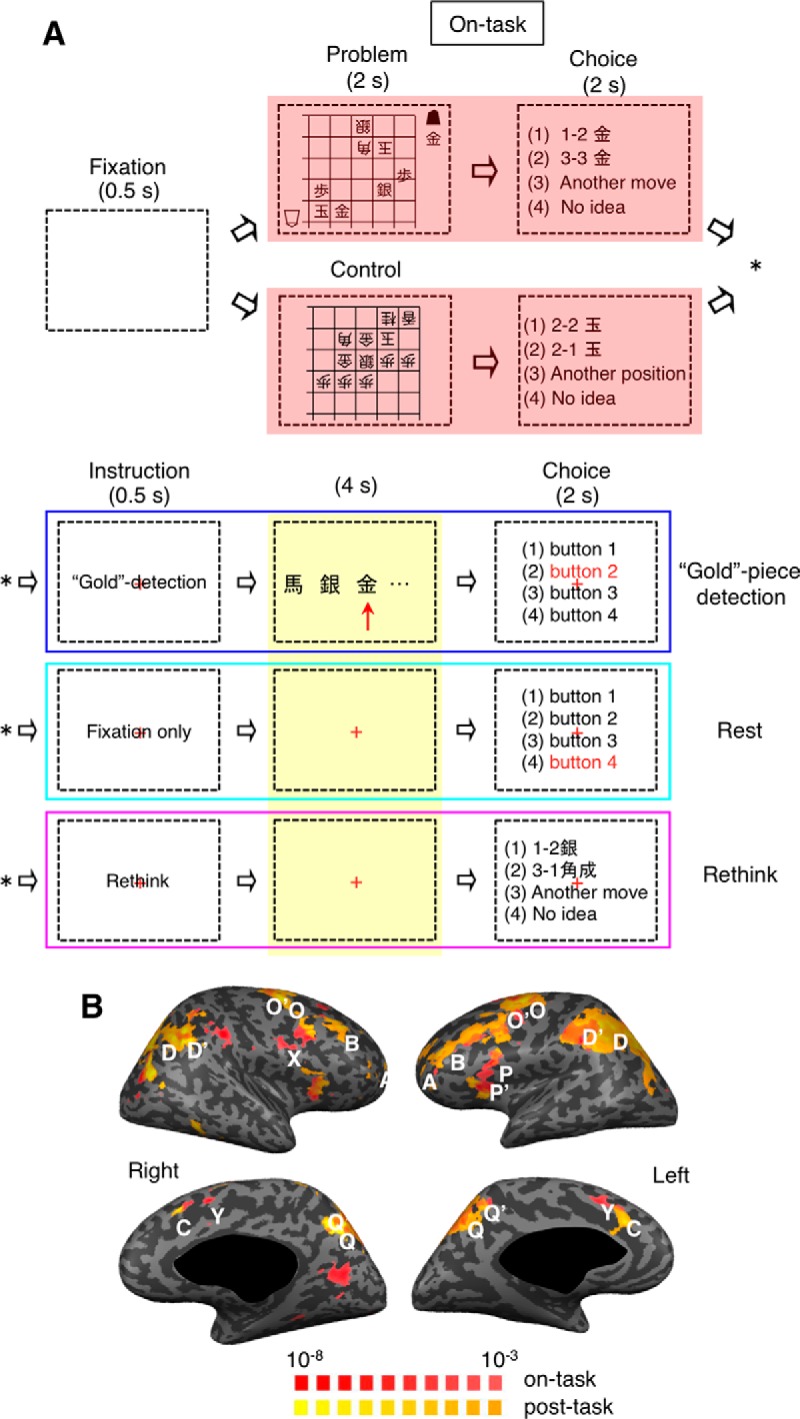
Tasks and activation patterns in Experiment 3. ***A***, Main task events in each trial. The ITI period was filled with “Gold” piece detection task, “rest,” or “rethinking” after the generation task, and with “Gold” piece detection task or “rest” after the control task. ***B***, Statistical parametric maps for the on-task activation (the on-task period contrasted with the post-task period in generation-task trials) and for the post-task activation (the post-task period after the generation task contrasted with the post-task period after the control task). The trials with “Gold” piece detection and with “rest” were combined. Those with “rethinking” were not included. Other conventions are the same as in Figure 1*B*.

According to the multiple-task switching hypothesis (Braver et al., 2003) for the function of the rostral frontal cortex, the post-task activation might be associated with the task switching between the next-move generation task and subsequent “Gold” piece detection task. To examine this possibility, we included the trials in which the subject was only required to maintain eye fixation during the ITI period (“rest” condition) in Experiment 3 (Fig. 8*A*; see Experiment 3). The board position was presented for 2 s, instead of 1 s, because only (high-level) amateur players were recruited in this experiment. There was no questionnaire, and we therefore commenced the 3.5 s post-task period at the offset of the 0.5 s instruction for the ITI condition in the GLM analysis.

**Figure 9.**
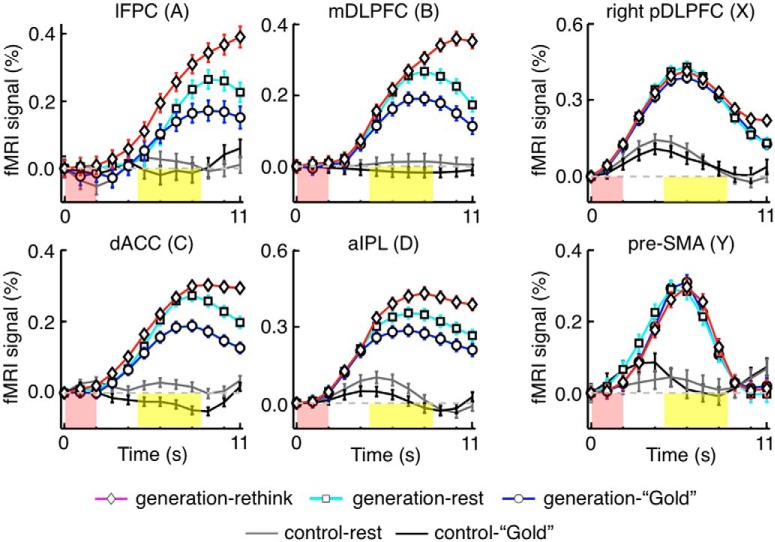
Activation time courses in Experiment 3. Signal changes were aligned to the onset of problem presentation. Error bars indicate SEM across trials. Red and yellow shadows represent the problem presentation and ITI periods, respectively. The differential effects from the previous generation and control trials were removed by subtracting the differences between mean signal changes in generation trials preceded by a generation trial and those in generation trials preceded by a control trial in Experiment 3 from the time courses of signal changes in individual trials preceded by a generation trial (see Adjusting time courses of BOLD signal changes in Experiments 1 and 3).

The same sets of cortical areas, respectively, exhibited on-task and post-task activations in Experiment 3 as those in Experiment 1 (Fig. 8*B*). Figure 9 shows mean time courses of BOLD signal changes. The initial declining trends in the trials following a generation trial had been removed as in Experiment 1 (see Adjusting time courses of BOLD signal changes in Experiments 1 and 3). The GLM analysis revealed that BOLD signal changes in the ROIs of the post-task network in the “rest” condition following the next-move generation task were significantly larger than those in the “rest” condition following the sensory-motor control task (two-tailed paired *t* test with 17 subjects, lFPC: *t*_(16)_ = 3.9, *p* = 0.0006; mDLPFC: *t*_(16)_ = 3.6, *p* = 0.001; dACC: *t*_(16)_ = 3.2, *p* = 0.003; aIPL: *t*_(16)_ = 3.3, *p* = 0.002; Fig. 9, left). They were also larger than those in the “Gold” piece detection condition following the generation task (two-tailed paired *t* test with 17 subjects, lFPC: *t*_(16)_ = 2.4, *p* = 0.015; mDLPFC: *t*_(16)_ = 2.2, *p* = 0.021; dACC: *t*_(16)_ = 2.0, *p* = 0.029; aIPL: *t*_(16)_ = 2.1, *p* = 0.026; Fig. 9, left). As expected, there were no significant differences between the two conditions in the two ROIs of the on-task network (two-tailed paired *t* test with 17 subjects, right pDLPFC: *t*_(16)_ = 1.1, *p* = 0.30; pre-SMA: *t*_(16)_ = 1.0, *p* = 0.36; Fig. 9, right).

These results suggest that the task switching was unlikely the cause for the post-task activation. They also demonstrated that the post-task activation did not crucially depend on the ongoing task during the ITI period, even though the engagement in the “Gold” piece detection partly reduced the post-task activation. In addition, because there was no questionnaire, either about confidence or memory at the beginning of the ITI period in Experiment 3, the cause of the post-task activation by these explicit evaluation processes is also excluded. Finally, the modulation of the post-task activation by the condition during the ITI period further supports our conclusion that the post-task activations were caused by neural processes that occurred after the preceding decision. If the neural processes that caused post-task activation had occurred before the onset of the ITI period, they could not have been modulated by the task condition during the ITI period. Thus, we contend that the post-task activations were caused by neural processes that occurred after, but in association with, the preceding decision.

There was little on-task activation in sensory-motor control trials in Experiment 3 (Fig. 9, right). The difference between the results in Experiments 1 and 3 was likely due to the difference in the presentation time of board position (1 s in Experiment 1 and 2 s in Experiment 3) and in the proportion of control trials (40% in Experiment 3 and 25% in Experiment 1). The subjects might always prepare a task set for the generation task in Experiment 1, whereas they prepared the task set after detecting that the presented board position was specific for the generation task (i.e., including own pieces) in Experiment 3.

### Association of the post-task activation with the preceding on-task activation

**Figure 10.**
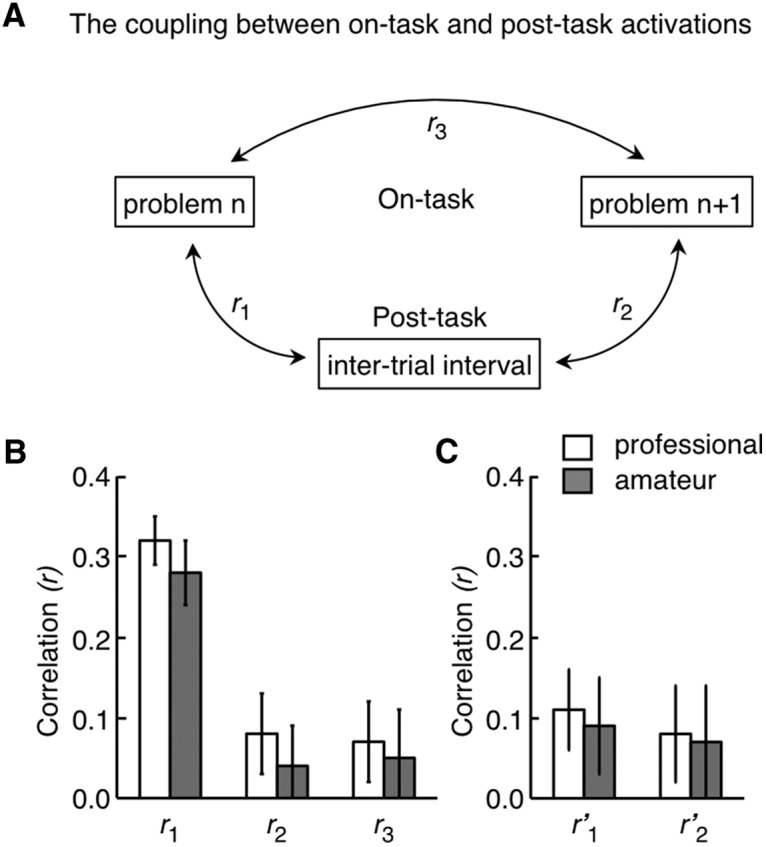
Correlation of trial-by-trial variations of BOLD signal changes. ***A***, The correlation was calculated between post-task activation in the post-task network and on-task activation in the on-task network associated with the preceding generation process (*r*_1_), between the post-task activation and the on-task activation associated with the subsequent generation process (*r*_2_), and between the on-task activations in the consecutive generation trials (*r*_3_). ***B***, Correlation coefficients in the three combinations. ***C***, Results of similar analyses applied to the post-task activation after the control task. Error bars indicate SEM across subjects.

Multiple lines of evidence so far have suggested that the post-task activation was associated with the preceding generation task. To further clarify this association, we analyzed the correlation of trial-by-trial variations of the post-task activation in the post-task network with those of the preceding on-task activation in the on-task network (Fig. 10*A*, *r*_1_). Alternatively, the post-task activation might influence the performance of the generation task in the next trial. We thus also analyzed the correlation of the post-task activation in the current trial with the on-task activation in the next trial (Fig. 10*A*, *r*_2_). The data from Experiment 1 were used for these analyses. We used pairs of consecutive generation trials in the analysis of prospective association (*r*_2_). For a fair comparison, we also focused on the first trials of two consecutive generation trials in the analysis of retrospective association (*r*_1_). The coefficients of the correlation were averaged across all ROI pairs between the two networks for each subject (see Analysis of functional connectivity between the on-task and post-task networks).

The strength of the post-task activation was significantly correlated with that of the preceding on-task activation (Fig. 10*B*, *r*_1_) (two-tailed, one-sample *t* test with 17 subjects, mean *r* = 0.32, *t*_(16)_ = 4.6, *p* = 1.5 × 10^−4^ for the professional group; two-tailed, one-sample *t* test with 17 subjects, mean *r* = 0.28, *t*_(16)_ = 4.3, *p* = 2.7 × 10^−4^ for the amateur group), but not with that of the on-task activation in the succeeding generation trial (Fig. 10*B*, *r*_2_) (two-tailed, one-sample *t* test with 17 subjects, mean *r* = 0.09, *t*_(16)_ = 1.2, *p* = 0.12 for the professional group; two-tailed, one-sample *t* test with 17 subjects, mean *r* = 0.04, *t*_(16)_ = 0.6, *p* = 0.28 for the amateur group). The former correlation was significantly larger than the latter (two-tailed paired *t* test with 17 subjects, *t*_(16)_ = 3.8, *p* = 7.3 × 10^−4^ for the professional group; *t*_(16)_ = 3.4, *p* = 0.0017 for the amateur group). Meanwhile, there was no significant correlation between on-task activations in consecutive generation trials (Fig. 10*B*, *r*_3_) (two-tailed, one-sample *t* test with 17 subjects, mean *r* = 0.07, *t*_(16)_ = 0.89, *p* = 0.20 for the professional group; two-tailed, one-sample *t* test with 17 subjects, mean *r* = 0.06, *t*_(16)_ = 0.78, *p* = 0.22 for the amateur group). Because there was also no significant correlation in either the retrospective direction in control trials (Fig. 10*C*, *r’*_1_; two-tailed, one-sample *t* test with 17 subjects, mean *r* = 0.11, *t*_(16)_ = 1.3, *p* = 0.10 for the professional group; two-tailed, one-sample *t* test with 17 subjects, mean *r* = 0.09, *t*_(16)_ = 1.1, *p* = 0.14 for the amateur group) or the prospective direction in sequences of a control trial followed by a generation trial (Fig. 10*C*, *r’*_2_; two-tailed, one-sample *t* test with 17 subjects, mean *r* = 0.08, *t*_(16)_ = 1.0, *p* = 0.17 for the professional group; two-tailed, one-sample *t* test with 17 subjects, mean *r* = 0.07, *t*_(16)_ = 0.91, *p* = 0.19 for the amateur group), the difference between the retrospective and prospective directions in generation-generation trial sequences could not be due to the difference in ITI. These results demonstrated that post-task activations in the post-task network, after the option selection, were clearly influenced by the preceding problem-solving process, but they did not have impact on the subsequent problem-solving process. As a final note, behaviorally, post-task activations were not correlated with either the response accuracy or the response time of the next generation trial (two-tailed, one-sample *t* test with 34 subjects, *r* = -0.06, *t*_(33)_ = 0.6, *p* = 0.27 for response accuracy; two-tailed, one-sample *t* test with 34 subjects, *r* = 0.05, *t*_(33)_ = 0.3, *p* = 0.39 for response time).

### Correlation of post-task activation with subject’s uncertainty about the preceding decision

**Figure 11.**
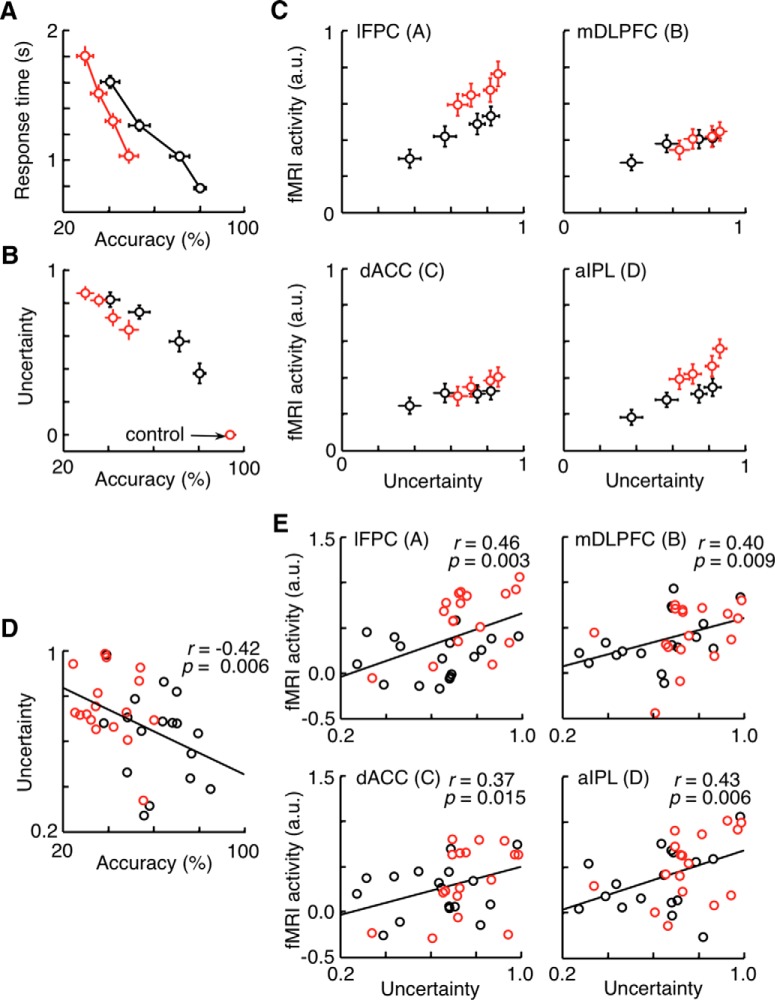
Correlation of post-task activations with uncertainty about the preceding decision in Experiment 1. Trials were divided into quarters in each subject according to the response time for ***A-C***. ***A***, The mean response time in each trial group plotted against the accuracy in the trial group (proportion of correct trials). Data points indicate the means in each subject group (professional or amateur). Error bars indicate SEM across subjects. ***B***, The mean degree of uncertainty in each trial group (proportion of trials with confidence report of “no”) plotted against the accuracy in the trial group. ***C***, Off-task activations in each trial group plotted against the mean degree of uncertainty in the trial group. ***D***, Overall degree of uncertainty of each subject plotted against the overall accuracy of the subject. ***E***, Mean off-task activations in each subject plotted against the overall mean degree of uncertainty of the subject. Black circles represent data from professional players. Red circles represent amateur players.

Given the retrospective nature of the correlation between post-task and on-task activations, we posit that post-task activations may be related to the subject’s uncertainty about the preceding problem-solving process. Although we obtained a binary report of the subject’s confidence level (yes or no) after a decision was made for each trial in Experiment 1, we used the response time to quantify the uncertainty level because the criteria for binary confidence reports appeared to largely vary among subjects. The proportion of the trials in which each subject gave a confident report did not correlate with the percentage of correct responses of the subject (two-tailed *t* test, *p* = 0.12 in the amateur group and *p* = 0.58 in the professional group). The 180 trials in the next-move generation task in Experiment 1 were divided into four equally sized groups for each subject according to the response time. The mean response time in a trial group was negatively correlated with the proportion of correct responses in the trial group (Fig. 11*A*): the regression coefficient determined for individual subjects was -0.42 ± 0.05 (mean ± SEM across subjects), which was significantly smaller than 0 (Model II regression, one-tailed one sample *t* test with 34 subjects, *t*_(33)_ = 3.36, *p* = 0.0008). The mean degree of uncertainty (the proportion of trials with report of “no”) in a trial group, on the other hand, was negatively correlated with the proportion of correct trials in the trial group (Fig. 11*B*): the regression coefficient determined for individual subjects was -0.48 ± 0.13 (mean ± SEM across subjects), which was significantly smaller than 0 (Model II regression, one-tailed one sample *t* test with 34 subjects, *t*_(33)_ = 3.69, *p* = 0.0004). Thus, the subjective confidence report reliably reflected the actual performance. In all four ROIs of the post-task network, we found that the post-task activations in a trial group were positively correlated with the degree of uncertainty in the trial group (Model II regression, two-tailed *t* test with 34 subjects, *p* values < 0.01; Fig. 11*C*). The association of the post-task activations with the uncertainty about the preceding decision was confirmed by the significantly larger post-task activations in the trials in which the subject gave an unconfident report than those in the trials in which the subject gave a confident report (two-tailed paired *t* test, *p* values < 0.01 in all four ROIs of the post-task network).

We also examined the across-subject correlation between mean post-task activations, averaged over all the trials for a subject, and the overall degree of uncertainty of the subject (proportion of the trials, in which the subject was not confident). Across subjects, the overall degree of uncertainty was highly correlated with the mean response accuracy (two-tailed, one-sample *t* test with 34 subjects, *r* = -0.42, *p* = 0.006; Fig. 11*D*). In all four ROIs of the post-task network, we found that the mean BOLD signal change in a subject was positively correlated with the subject’s overall degree of uncertainty (two-tailed, one-sample *t* test with 34 subjects, *p* < 0.01; Fig. 11*E*).

When the same analyses were applied to on-task activations in the two areas of the on-task network, there was no significant correlation between on-task activations and the subject’s degree of uncertainty. Both the across-trial correlation within individual subjects and the across-subject correlation were not significant in either area (two-tailed *t* test with 34 subjects, *p* > 0.20 for the across-trial correlation and *p* > 0.26 for the across-subject correlation in both ROIs). However, there was a possible pitfall in this analysis; that is, the longer on-task-period regressor in the GLM may have diluted on-task activations associated with slow (and unconfident) responses. We thus conducted another GLM analysis, in which a fixed-duration on-task-period regressor, made by convolving the mean response time in the subject with the canonical HRF, was used to calculate the magnitude of on-task activation. This analysis allowed us to detect a marginal positive correlation between on-task activations and the uncertainty in individual subjects (pDLPFC, Model II regression, two-tailed, one-sample *t* test with 34 subjects, *t*_(33)_ = 1.56, *p* = 0.064; pre-SMA, *t*_(33)_ = 1.62, *p* = 0.057), suggesting that neural activations in the on-task network may also reflect the subject’s uncertainty.

### Involvement of the post-task network in decision adjustment

**Figure 12.**
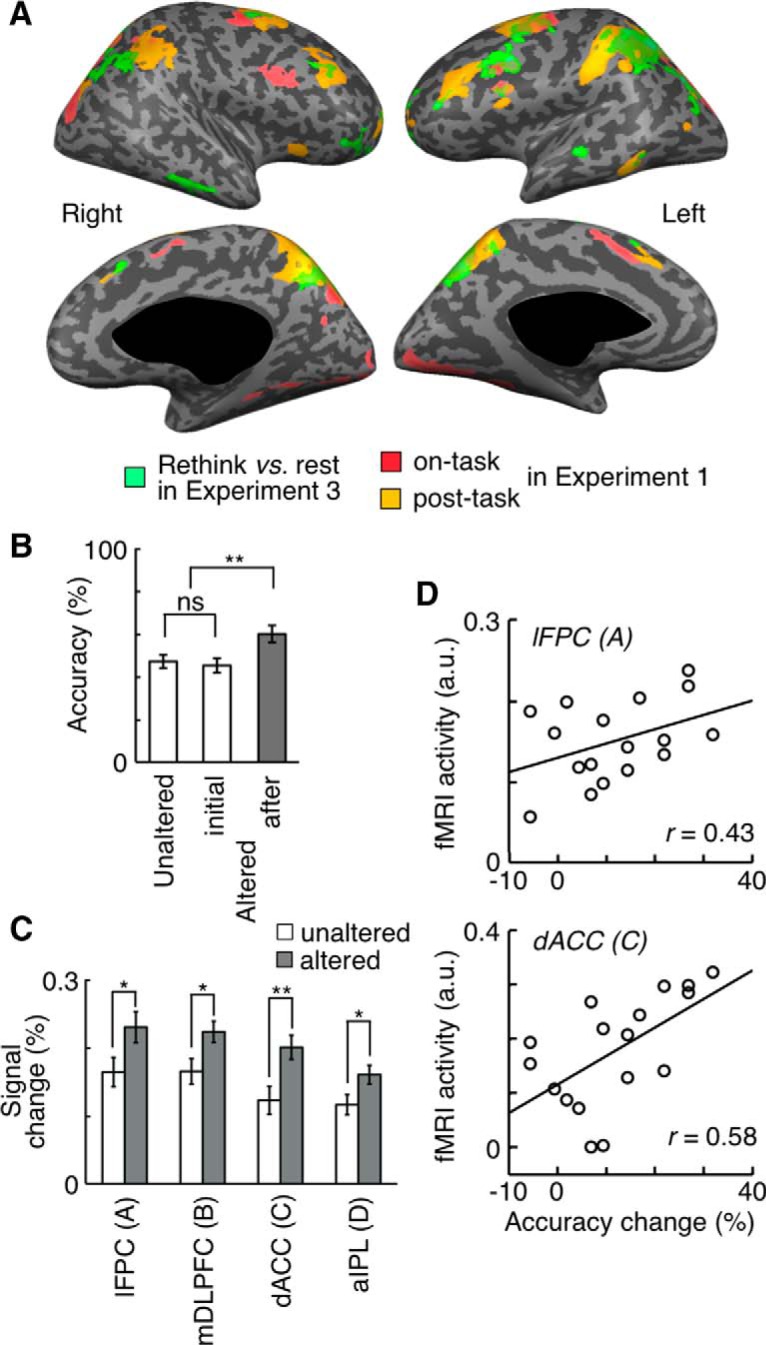
Activations associated with rethinking and correlation of activation magnitude with accuracy improvement in Experiment 3. ***A***, Brain regions that increased activities during the post-task period by rethinking the preceding problem compared with the activities at rest during the post-task period after the generation task (green). Regions displayed in Figure 4*A* for on-task activation (red) and post-task activation (yellow) are shown again for comparison. Regions activated by rethinking largely coincided with post-task activations (*p* < 0.05, FDR corrected). ***B***, The mean accuracy in the trials in which the subject did not alter the selection (left), the mean accuracy of the first answers (middle), and the mean accuracy after rethinking (right) in the trials in which the subject altered the selection. ***C***, Rethinking-associated activations in unaltered trials (open bars) and altered trials (gray bars). ***B***, ***C***, *, *p* < 0.05. **, *p* < 0.01. ns, Not significant. Error bars indicate SEM across subjects. ***D***, The magnitude of rethinking-associated activation in each subject plotted against the mean accuracy improvement of the subject by rethinking: *r* = 0.43 and *p* = 0.04 for lFPC, and *r* = 0.58 and *p* = 0.007 for dACC.

Experiment 3 contained an additional condition for the ITI period, in which the subject was instructed to rethink the preceding next-move problem (Fig. 8*A*). We contrasted BOLD signal changes when the subject was engaged in “rethinking” with those when the subject merely maintained fixation (i.e., “rest”) during the post-task period following the generation task. Activated cortical areas that were identified in this comparison coincided to a large extent with the areas activated during the post-task period after the generation task, but not with those activated during the generation task in Experiment 1 (Fig. 12*A*). This specific augmentation of post-task activations by rethinking was also confirmed by ROI analyses for the ROIs determined in Experiment 1. Activations during rethinking were stronger than post-task activations in rest and “Gold” piece detection conditions in all four ROIs of the post-task network (two-tailed paired *t* test with 17 subjects, lFPC: *t*_(16)_ = 2.8, *p* = 0.012; mDLPFC: *t*_(16)_ = 2.6, *p* = 0.020; dACC: *t*_(16)_ = 2.6, *p* = 0.020; aIPL: *t*_(16)_ = 2.5, *p* = 0.024; Fig. 9, left). In contrast, rethinking did not activate the areas in the on-task network: β values for the post-task period regressor in the rethinking condition were not different from those in either the rest or the “Gold” piece detection condition (two-tailed, one-sample *t* test with 17 subjects, right pDLPFC: *t*_(16)_ = 0.9, *p* = 0.38; pre-SMA: *t*_(16)_ = 0.8, *p* = 0.44) and β values for the on-task period regressor in the rethinking condition were not different from those in either the rest or the “Gold” piece detection condition (two-tailed, one-sample *t* test with 17 subjects, right pDLPFC: *t*_(16)_ = 0.8, *p* = 0.44; pre-SMA: *t*_(16)_ = 0.7, *p* = 0.46) (Fig. 9, right). These results show that the post-task network rather than on-task network was recruited to rethink the preceding problem to which the subject had once responded.

The option selection was altered by rethinking in about half of the trials (52 ± 4%, mean ± SEM), and the response accuracy was improved by the alteration: the accuracy after alteration was significantly higher than those in the first thinking (two-tailed paired *t* test with 17 subjects, *t*_(16)_ = 2.7, *p* = 0.0081) and in unaltered trials (two-tailed paired *t* test with 17 subjects, *t*_(16)_ = 2.8, *p* = 0.0059; Fig. 12*B*). Furthermore, when activations during rethinking in the post-task network were compared between altered and unaltered trials, those in altered trials were found to be stronger in all four ROIs of the post-task network (two-tailed paired *t* test with 17 subjects, *p* < 0.05 for all four areas; Fig. 12*C*). We also found that the accuracy change by rethinking for each subject was significantly correlated with activations during rethinking in the subject’s lFPC (two-tailed, one-sample *t* test with 17 subjects, *r* = 0.43, *p* = 0.04; Fig. 12*D*, top) and dACC (two-tailed, one-sample *t* test with 17 subjects, *r* = 0.58, *p* = 0.007; Fig. 12*D*, bottom), and marginally with those in mDLPFC (two-tailed, one-sample *t* test with 17 subjects, *r* = 0.34, *p* = 0.08) and aIPL (two-tailed, one-sample *t* test with 17 subjects, *r* = 0.35, *p* = 0.08). In brief, these results demonstrated that activations during rethinking in the post-task network were correlated with the beneficial consequence of rethinking, both across trials in individual subjects and across subjects.

### Increased post-task activation as a result of training

**Figure 13.**
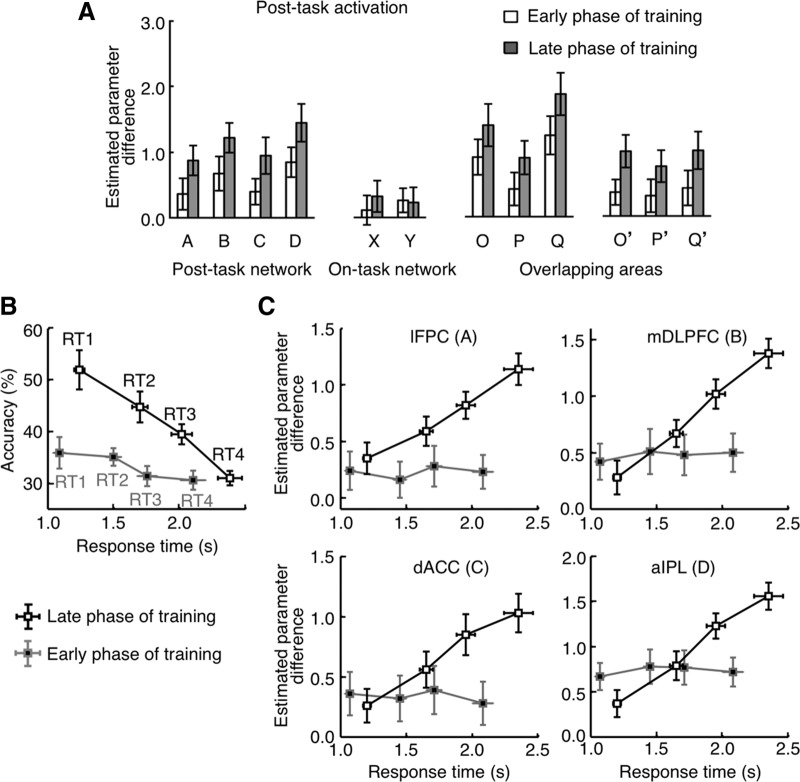
Changes of post-task activations in originally novice subjects along a long-term training in Experiment 4. ***A***, Bars represent differences between β values determined by GLM analyses for the post-task period regressor in generation-task trials and those determined for the post-task period regressor in control-task trials. Open and gray bars represent the results obtained in an early phase (second or third week) and at a late phase (the last week), respectively, of a long-term (15 weeks) training. Error bars indicate SEM across subjects. ***B***, Response accuracy in each of the four trial groups plotted against the mean response time in the trial group at the early and late phases of training. ***C***, Post-task activations in each of the four trial groups plotted against the mean response time in the trial. ***B***, ***C***, Trials were divided into quarters in each subject according to the response time. The data points indicate the mean values averaged over the 19 subjects. Error bars indicate SEM across subjects. Gray and black lines indicate the values at the early and late phases of training, respectively.

In Experiment 4, 19 subjects, who had had no prior experience of playing shogi, learned and practiced daily to play games of a simplified shogi (gogo-shogi) for 15 weeks (Tasks: Experiment 4). Brain activities associated with the quick-generation task (with 2 s board position presentation) were examined twice, at the early (the 2-3 weeks) and end (the 14-15 weeks) phases of the training. We found that post-task activations in the post-task network increased significantly from the first to the second measurement (two-tailed paired *t* test with 19 subjects, *p* < 0.05 in all areas; Fig. 13*A*), whereas on-task activations in the on-task network did not change (Wan et al., 2012, their Fig. 4).

When trials were divided into quarters in each subject according to the response time, the mean accuracy (percent of correct responses) in each trial group was negatively correlated with the mean response time of the trial group at the late phase of the training (regression coefficient was -0.66 ± 0.19, mean ± SEM across subjects, model II regression, which were significantly less than 0, two-tailed, one-sample *t* test, *t*_(18)_ = 3.49, *p* = 0.001), but not at the early phase of the training (regression coefficient was -0.10 ± 0.30, mean ± SEM, model II regression, two-tailed, one-sample *t* test, *t*_(18)_ = 0.34, *p* = 0.37; Fig. 13*B*). Concurrently, the magnitude of post-task activations of each area of the post-task network in each trial group was correlated with the mean response time of the trial group only at the late phase of the training (*p* < 0.01 in any of the ROIs; Fig. 13*C*), but not at the early phase of the training (*p* > 0.10 in any of the ROIs, Fig. 13*C*).

## Discussion

### Late-onset BOLD responses caused by postdecision neural activations

By measuring brain activities of experienced players while they were solving complex rule-based problems, the checkmate problems of shogi, we revealed that a frontoparietal network composed of rostral frontal cortical regions, including lFPC, mDLPFC, and dACC, along with aIPL, was activated only during the post-task period of a few seconds after the subjects generated the ideas of the best next-move. This post-task activation appeared after quick intuitive generation as well as after deliberate search, but not after performing a sensory-motor control task. By virtue of large variation of the on-task duration in Experiment 2, we confirmed that the responses were aligned to the end of the generation task, or the onset of ITI, but not to the beginning or the middle period of the generation task. That is, fitting of BOLD responses with different models demonstrated that the late-onset BOLD responses were caused by postdecision neural activations that occurred immediately after the preceding decision, but not by delayed hemodynamic responses coupled with neural activations that occurred during the on-task period. This inference was further supported by the observation that the magnitude of the post-task activation was modulated by the condition during the ITI period in Experiment 3.

While the post-task activations were caused by neural activations that occurred after the completion of the preceding decision, several lines of evidence suggest that those neural activations were associated with the execution of the preceding generation task, but not the ongoing task during the post-task period. First, the post-task activation did not occur after performing the sensory-motor control task. Second, trial-by-trial variations of the post-task activation were correlated with those of the activation that occurred in another set of brain areas during the preceding on-task period. Third, trial-by-trial variations of the post-task activation were correlated with the subjects’ degree of uncertainty about the correctness of the preceding decision. On the other hand, the ongoing task during the post-task period had only a modulatory influence on the post-task activation. The “Gold” piece detection task, which was devised to interrupt the subject’s thinking about the problem given in the preceding generation task, reduced the post-task activation by only 20%-40% compared with that during fixation only.

Little attention has been paid to the postdecision processing in fMRI task paradigms in which there was no explicit feedback, such as a reward or an error/correct signal. Although late-onset BOLD responses were previously observed in lFPC after the familiar-novel decision on visually presented words, the nature of neural activations that caused the late-onset BOLD responses was not determined in previous studies (Schacter et al., 1997; Buckner et al, 1998; Reynolds et al., 2006). Activation of the default-mode network during rest, compared with the activity during task periods, has been repeatedly demonstrated, but the default-mode activation is different from the post-task activation found in the present study, in that the default-mode activation does not depend on the preceding task (Raichle et al., 2001; Buckner et al., 2008). Indeed, there was little overlap between the default-mode and post-task networks. For example, within the frontopolar cortex, the medial part has been assigned as a part of the default-mode network, whereas the lateral part belonged to the post-task network. A network similar to the post-task network has been identified by analyzing the functional connectivity during rest (Vincent et al., 2008), but functions of the network have been little explored (but see Cole et al., 2013).

By meta-analyzing a large set of human imaging studies, it has been shown that many different cognitive demands recruit three broad, yet confined, regions of the prefrontal cortex: the dorsal part of anterior cingulate region, the mid-dorsolateral region around the middle and caudal parts of the inferior frontal sulcus, and the mid-ventrolateral region extending from the frontal operculum to the anterior insula (Duncan and Owen, 2000). This set of regions was named as the multidemand system and discussed to be critical for the identification of subtasks and control of their sequential recruitment to achieve remote goals (Duncan, 2010). While each of the three regions was elongated widely in the rostrocaudal dimension, the functional gradient within the system has not been discussed. The regions of the post-task network and those of the on-task network were located in the rostral and caudal parts of the mid-dorsolateral and dorsal anterior cingulate regions of the multidemand system. Thus, our current study indicates a functional subdivision within the multidemand system. The post-task network partly overlapped with both the frontoparietal and cingulo-opercular networks of Dosenbach et al. (2006, 2007,2008).

### Properties of the postdecision activation

As there was no explicit feedback after the preceding decision in the present study, the postdecision activations could not represent the outcome expectation error. The correlation of trial-by-trial variations of the postdecision activation with the subjective uncertainty about the preceding decision suggests that it is the uncertainty about the preceding decision that triggered the postdecision neural activations. Because explicit rethinking of the preceding problem activated the same network, the postdecision activations should contain functional components that overlap with those of rethinking. Thus, it is likely that the postdecision activations observed in our study represented the evaluation and adjustment procedures. Except for the rethinking condition, there was no explicit task requirement or merit for these procedures. The evaluation and adjustment procedures might automatically occur in experienced players as they help the players to better understand the game. Indeed, the postdecision activations were absent in the subjects who had just started to play the game of shogi in less than 3 weeks but emerged after the subjects underwent extensive daily training for 4 months (Experiment 4).

### Proposition of the post-task network’s general roles in strategy management

Frontal areas in the post-task network, including lFPC, are activated while the subjects perform tasks of higher-order structure or abstract information processing (Baker et al., 1996; Koechlin et al., 1999; Christoff et al., 2001; Ramnani and Owen, 2004; Koechlin and Summerfield, 2007; Badre and D’Esposito, 2007, 2009). lFPC and dACC are activated during uncertainty-driven exploration (Daw et al., 2006; Boorman et al., 2009, 2013; Badre et al., 2012; Kolling et al., 2012, 2014), and by a metacognitive process to report the confidence level in visual memory (Yokoyama et al., 2010) and in perceptual judgment of noisy images as well (Fleming et al., 2012). The task of rethinking of the preceding problem, which activated the post-task network in the present experiment, and the tasks used in the previous studies described above have overlapping components. Rethinking of the preceding problem includes evaluation of the preceding decision and exploration of alternative moves. Exploration of alternatives and execution of tasks with higher-order structure require meta-level monitoring of multiple processes. Uncertainty monitoring and exploration of alternatives are the two key components of the metacognitive function (Nelson and Narens, 1990). Thus, here we propose that the post-task network, or the frontoparietal network, mediates a metacognitive control process for monitoring and adjusting decision-making and learning strategies.

Generation of the best next-move for a given board position is thought to comprise a series of complex cognitive processes, including recognizing the position, selecting a particular problem-solving strategy, generating sequences of moves that reach the goal, and selecting the best move sequence (de Groot, 1965; Newell and Simon, 1972; Zelazo et al., 1997). Such a complicated process may recruit a metacognitive control process. However, the subjects who participated in the present study were either professional or experienced amateur players. As extensive training on the game of shogi makes the process automatic and the automated process may be implemented in the caudal frontal regions, the frontoparietal network is likely recruited only during the post-task evaluation and adjustment. In other words, whether the frontoparietal network works in the postdecision stage alone or in the on-task control as well may depend on the nature of the task to be performed and the subject’s experience with the task.

In conclusion, our findings suggest that recruitment of cognitive control in the frontal cortex is subject to the strategy of task implementation. The caudal frontal areas mainly control the default strategy of exploiting routine processes, whereas the frontoparietal network, including the rostral frontal areas, mainly controls exploration of alternative processes. Our findings also indicate that the exploitation in the caudal frontal areas and the exploration in the frontoparietal network may work in the same task in a complementary manner: the exploitation works during online task execution, whereas the exploration works during postdecision evaluation and adjustment.

## Synthesis

The decision was a result of the Reviewing Editor Philippe Tobler and the peer reviewers coming together and discussing their recommendations until a consensus was reached. A fact-based synthesis statement explaining their decision and outlining what is needed to prepare a revision is listed below. The following reviewers agreed to reveal their identity: Akira O'Connor, Sabina Gherman

Dear Dr. Tanaka,

Your manuscript has now been seen by two reviewers. Their comments are combined below. They found merit but make several constructive remarks that need to be addressed in a revision, which I look forward to receiving.

Best regards,

Philippe Tobler

The authors report results from 4 experiments, aiming to demonstrate that post-decision evaluation following a task that requires making decisions on Japanese chess problems is supported by activation in a fronto-parietal network. They present evidence that activation in this network: 1) is unique to the post-task period (i.e., not present during task performance itself), yet is related to (i.e., influenced by) activation during the preceding task; 2) can be observed after the problem solving task but not after a sensori-motor control task; 3) is enhanced when subjects are prompted to "rethink" their choice during the post-task period, compared to when they perform a distraction task (additionally, changing choice after rethinking improves performance); 4) correlates with uncertainty of choice in the preceding problem solving task; 5) is enhanced in novice subjects after extensive training. They conclude that the identified network may play a role in post-decision uncertainty monitoring and/or exploration of alternatives.

The tasks used are relatively unusual for the literature (shogi check-mate problems) but are far superior to many decision-making tasks in that they require participants to have undergone many hundreds of hours of training and are tasks in which the expert participants are extremely heavily invested. The manuscript has been revised to consider two previous reviewers' points and this appears to have been carried out as comprehensively as could be expected. I have a small number of additional minor points which the authors may wish to deal with in any future revision.

Taken collectively the results are interesting and make for a compelling argument. Overall, the authors perform rigorous control analyses to rule out alternative explanations for their main findings. There are some methodological concerns that should be clarified and/or addressed:

1. Details of the GLM analysis on the fMRI data are at times unclear (or incomplete). In the Methods section, authors state that all regression analyses used only two regressors (one for the on-task period and one for the post-task period). Firstly, it is not clear how the different conditions (main task vs. control task, type of ITI etc.) were modelled and how follow-up comparisons between conditions or subsets of trials were conducted (for example between [ON-TASK main task trials vs. POST-TASK main task trials], or between [ITI control trials vs. ITI main task trials]* (Experiment 1, Fig. 4B). Secondly, authors do not mention whether they modelled out potential confounding task-related factors (e.g., visual stimulation, onset/duration of different ITI events, motor response etc.) in their GLM analyses.

(*Note: I use "main task" to refer to the problem solving, i.e. next-move generation, condition)

2. Authors choose to use response times (RTs) as an index of uncertainty (despite collecting confidence reports in Experiment1), however they do not provide an explicit justification for this choice. RT is indeed known to correlate with confidence thus making it a good alternative measure when confidence reports are not available. Also, I can see how using a graded measure like RTs has an advantage over the binary confidence reports when demonstrating the association with post-task activation. However, it would be useful to confirm that post-task activation in the post-task ROIs is also modulated by subjective confidence (i.e., a High vs. Low contrast).

Minor comments/suggestions

1.Not being familiar with the game, I would appreciate an additional sentence explaining whether check-mate problems are problems in which the participant is required to achieve a check-mate (which I assume from the move-limited way in which the check-mate is described) or avoid a check-mate.

2.The abstract may benefit from additional restructuring as it feels a little fragmented (for example the part "while the responses were caused by post-decision neural activations" can probably be omitted; also, it might help to move the conclusion that "post-decision neural activations were associated with the preceding decision procedure" to after all evidence has been mentioned (that is, after "absence of responses after performing a sensory-motor control task" and "the magnitude of post-decision activations was correlated with the degree of uncertainty").

3.The authors write: "The on-task period started at the onset of the problem presentation, and in Experiments 1, 3 and 4, it included the entire time for problem presentation and the difference in response time, obtained by subtracting the mean response time of the subject in the sensory-motor control task from the response time in individual trials." (Methods, Determination of post-task period and associated ROIs) > From the text it is not clear why the on-task period was defined as it was (my only guess is that it was done in an effort to limit motor preparation activation, however this is not explained)

4."studies have found evidences" (Introduction, 1st paragraph) > evidence

5."the piece of King" (Methods, Experiment 1) > the "King" (piece)

6."As there was no piece of the own side" (Methods, Experiment 1) > As there were no pieces on the participant's side

7."Differed from Experiments 1 and 2" (Methods, Experiment 3) > Unlike Experiments 1 and 2

8."Fig. 1A yellow patches" (Results, fMRI responses during the off-task or post-task period, 3rd paragraph) > Fig. 1B

9."and then we commenced" (Results, Robust post-task activation regardless of conditions during ITI, 1st paragraph) > additionally, we commenced

10."but in associated with" (Results, Robust post-task activation regardless of conditions during ITI) > but were associated with

11.Table 2 > It would be helpful to add a notation on the vertical axis to mark the on-task vs. post-task networks.

12."All the subjects were right-handed Japanese male." p.4. Consider "males"

13."The main results of Experiment 4 had been reported (Wan et al., 2012)." p.4. Consider "have been previously reported"

14."proceeded to an intertrial-interval (ITI) period, which were occupied with two simple..." p.4. Consider "proceeded to an intertrial-interval (ITI) period, during which subjects completed two simple..."

15.The "interruption task" term could be replaced with the more standard "distracter task" terminology.

16."each pair of a ROI in" p.4. Consider "each pair of ROIs in"
